# Computational Investigation of Bending Properties of RNA AUUCU, CCUG, CAG, and CUG Repeat Expansions Associated With Neuromuscular Disorders

**DOI:** 10.3389/fmolb.2022.830161

**Published:** 2022-04-11

**Authors:** Amirhossein Taghavi, Ilyas Yildirim

**Affiliations:** ^1^ Department of Chemistry and Biochemistry, Florida Atlantic University, Jupiter, FL, United States; ^2^ Department of Chemistry, The Scripps Research Institute, Jupiter, FL, United States

**Keywords:** RNA repeat expansion, MD simulation, AUUCU, CCUG, CUG, CAG, computational chemistry

## Abstract

Expansions of RNA AUUCU, CCUG, CAG, and CUG repeats cause spinocerebellar ataxia type 10, myotonic dystrophy type 2, Huntington’s disease, and myotonic dystrophy type 1, respectively. By performing extensive molecular dynamic simulations, we investigated the bending propensities and conformational landscapes adopted by 3×3, 2×2, and 1×1 internal loops observed in RNA AUUCU, CCUG, CAG, and CUG repeat expansions using model systems having biologically relevant repeat sizes. We show that the conformational variability experienced by these loops is more complex than previous reports where a variety of unconventional hydrogen bonds are formed. At the global scale, strong bending propensity was observed in r(AUUCU)_10_, r(CCUG)_15_, r(CAG)_20,_ and r(CUG)_20,_ and, to a lesser extent, in r(AUUCU)_4_, r(CCUG)_10_, r(CAG)_10_, and r(CUG)_10_. Furthermore, RNA CAG repeats exhibit a tendency toward bent states with more than 50% of observed conformations having bending angles greater than 50°, while RNA CUG repeats display relatively linear-like conformations with extremely bent conformations accounting for less than 25% of the observed structures. Conformations experienced by RNA AUUCU repeats are a combination of strongly bent and kinked structures. The bent states in RNA CCUG repeats mostly fall into the moderately bent category with a marginal ensemble experiencing extreme bending. The general pattern observed in all the bent structures indicates the collapse of the major groove width as the mechanical trigger for bending, which is caused by alteration of base pair step parameters at multiple locations along the RNA due to local distortions at the loop sites. Overextension is also observed in all the RNA repeats that is attributed to widening of the major groove width as well as undertwisting phenomenon. This information and the rich structural repository could be applied for structure based small molecule design targeting disease-causing RNAs. The bending propensities of these constructs, at the global level, could also have implications on how expanded RNA repeats interact with proteins.

## Introduction

Expanded RNA repeats are responsible for a wide range of neuromuscular diseases such as spinocerebellar ataxia type 10 (SCA10) (Matsuura et al., 2000; Teive et al., 2011), myotonic dystrophy type 2 (DM2) (Liquori et al., 2001), Huntington’s disease (HD) (Lee et al., 2012), and myotonic dystrophy type 1 (DM1) (Brook et al., 1992). Expanded RNA AUUCU repeats, r(AUUCU)^exp^, within intron 9 of the ATXN10 pre-mRNA causes SCA10, a disease with no cures available (Lin and Ashizawa, 2003; Ashizawa et al., 2006). Expanded RNA r(CCUG), r(CUCG)^exp^, residing within the intron 1 of the zinc finger 9 (ZNF9) precursor mRNA (pre-mRNA) cause DM2. Expansion of RNA CUG, r(CUG)^exp^, and CA repeats, r(CAG)^exp^, cause DM1 and HD, respectively. These expanded RNA repeats fold into structures, which can strongly interact with proteins like heterogeneous nuclear ribonucleoprotein K (hnRNP K) (White et al., 2010) in SCA10 and muscleblind-like 1 protein (MBNL1) in DM1, DM2, and HD (Mankodi et al., 2001; Fardaei et al., 2002). hnRNP K regulates splicing of *β*-tropomyosin (Bomsztyk et al., 2004; Wang et al., 2020) and its interaction with expanded RNA AUUCU repeats inactivate them, which ultimately causes cell death. The same mechanism holds true for inactivation of MBNL1 protein resulting in splicing defects. While r(CUG)^exp^ and r(CAG)^exp^ have repeating 1×1 UU and 1×1 AA internal loops, respectively, r(AUUCU)^exp^ and r(CCUG)^exp^ possess continuous 3×3 UCU/UCU and 2×2 CU/UC internal loops, respectively, with non-canonical UU, CC, and UC base pairs. As a result, they are highly dynamic and impart far more instability compared with non-canonical 1×1 internal loops.

Inhibition of RNA-protein complexes causing neuromuscular diseases, and hence, restoring cell regulation is a new therapeutic strategy, which has attracted a lot of attention (Bomsztyk et al., 2004; Wang et al., 2020). Among different methods adopted to target RNA for therapeutics, such as peptides and antisense nucleotides (ASOs) (van Roon-Mom et al., 2018), small molecules have the unique ability to target different RNA motifs found within RNA structures (Childs-Disney et al., 2014; Yang et al., 2016). While ASOs target the RNA based on the sequence complementarity, small molecules recognize specific RNA loop motifs observed within the conformational ensemble of RNA (Disney and Angelbello, 2016; Scoles et al., 2017), which can improve specificity in targeting RNA molecules. As a result, detailed investigation of RNA loops can provide the necessary data to help design drugs for pharmacotherapies.

Efficient exploration of the chemical space of small molecules targeting RNA requires deep understanding of the conformational variability adopted by RNA molecules. The 3×3 UCU/UCU and 2×2 CU/UC internal loops observed in r(AUUCU) and r(CCUG) repeats create challenges while studying the conformational landscapes experienced by these RNA repeats. In the meantime, however, due to their distinct nature, 3×3 UCU/UCU and 2×2 CU/UC internal loop motifs provide unique druggable targets, which could be exploited for therapeutic use. Despite the recent advancements in studying RNA dynamics using NMR (Liu et al., 2021) or cryo-EM (Bonilla et al., 2021), capturing the conformational variability observed in these complex RNA internal loops without contributions from computational techniques is very challenging. Thus, computational studies along with experimental studies can provide the necessary information at atomistic levels. It should be mentioned that in the absence of experimental data, computational studies are the only available tool that can provide atomistic insight on the conformational plasticity of RNA expanded repeats, although the limitations of currently available RNA force fields should be taken into consideration.

RNA dynamics plays an important role in defining its function, especially in RNA-protein interactions (MacRae et al., 2007; van Kouwenhove et al., 2011; Uchikawa et al., 2016; Hur, 2019). Although there are some studies exploring the dynamics of RNA internal loops in RNA repeat expansions (Yildirim et al., 2013; Yildirim et al., 2015), there are no detailed computational studies investigating the global structural properties of RNA repeats having biologically relevant repeat sizes. In DM1, DM2, and HD, the expended repeats have over 50 copies of the repeat, while in SCA10 expanded repeats are observed to have between 500 and 4,500 copies of AUUCU (Orr and Zoghbi, 2007). In this contribution, we utilized computational methods to investigate the bending properties of RNA AUUCU, CCUG, CAG, and CUG repeats, and atomic details of RNA 3×3 UCU/UCU, 2×2 CU/UC, 1×1 A/A, and 1×1 U/U internal loops observed in r(AUUCU)^exp^, r(CCUG)^exp^, r(CAG)^exp^, and r(CUG)^exp^ using biologically relevant RNA models (Ciesiolka et al., 2017). Three RNA models with different repeat sizes for each RNA repeat were prepared, where the most expanded models studied were 10×AUUCU, 15×CCUG, 20×CAG, and 20×CUG (Supplementary Figure S1). Extensive explicit solvent molecular dynamics (MD) simulations were performed on each system to investigate the conformational variability adopted by each RNA internal loop as well as the bending propensities of RNA models as a measure to determine the global structural characteristics of RNA repeats. Strong bending was observed in 10×AUUCU, 15×CCUG, 20×CAG, and 20×CUG and, to a lesser extent, in 4×AUUCU, 10×CCUG, 10×CAG, and 10×CUG. Strong kink structures were also observed in 10×AUUCU. RNA CAG repeats exhibited a tendency toward bent states due to the bulky 1× 1 A/A mismatches. Collapse of major groove width was found to be the common feature in the bent structures. Distortions at the RNA loop sites due to weak pairings alter local structural properties such as base pair step parameters, where multiple such local distortions when combined transform RNA to a bent or kinked geometries. Furthermore, overextension of RNA structures was observed that is attributed to widening of the major groove width as well as undertwisting. The results are important in structure-based drug design targeting RNA repeats as well as understanding how proteins interact with RNA repeats *via* bending phenomenon.

## Methods

### System Preparation

In order to explore the dynamics of expanded RNA repeats associated with neuromuscular diseases, at both local and global structural levels, we prepared a total of 12 systems (Table 1). Each system incorporating 1×1, 2×2 and 3×3 internal loops were prepared with three different repeat sizes. The NAB (Macke and Case, 1997) module of AMBER 16 was utilized to build the initial structures in A-form RNA orientations for each system. MD simulations were carried out with the AMBER 16 (Case et al., 2016) simulation package using the PARM99 force field (Cornell et al., 1995) with revised *χ* (Yildirim et al., 2010) and *α*/*γ* (Wales and Yildirim, 2017) torsional parameters. Each system was first neutralized with Na^+^ ions, (Joung and Cheatham, 2008), which then was solvated with TIP3P water molecules (Jorgensen et al., 1983) in a truncated octahedral box with periodic boundary conditions extended to 10 Å using the LEAP module of AMBER 16. Extra Na^+^ and Cl^−^ ions were added to each system to mimic the physiological conditions, where after equilibration the biggest RNA systems had ∼0.07 M Na^+^ concentrations while mid-sized RNA systems had around 0.12–0.29 M Na^+^ concentrations (Table 1). Addition of up to 0.25 M Na^+^ has been shown to not affect DNA bending implying that the bending phenomenon we studied using different sizes of RNA are comparable (Fields et al., 2013).

**TABLE 1:** RNA models utilized to investigate r(AUUCU)^exp^, r(CUCG)^exp^, r(CUG)^exp^, and r(CAG)^exp^.

Model system	Short name	MD simulation time (µsec)	System size^a^ (K)	Na^+^ concentration (M)
C(AUUCU)_2_AUC	2×AUUCU	90	22	0.42
C(AUUCU)_4_AUC	4×AUUCU	26	55	0.29
C(AUUCU)_10_AUC	10×AUUCU	2.9	331	0.08
(CCUG)_4_	4×CCUG	37	27	0.37
(CCUG)_10_	10×CCUG	5.6	148	0.12
(CCUG)_15_	15×CCUG	1.8	409	0.07
GG(CAG)_2_CC	2×CAG	100	14	0.44
GG(CAG)_10_CC	10×CAG	7.7	117	0.14
GG(CAG)_20_CC	20×CAG	2.1	480	0.06
GG(CUG)_2_CC	2×CUG	100	14	0.44
GG(CUG)_10_CC	10×CUG	7.6	117	0.17
GG(CUG)_20_CC	20×CUG	2.6	480	0.07

^a^System size represents total number of atoms including water and ions, where K stands for thousand. Note that sections underlined in each RNA sequence represent internal loops observed in these RNA repeats.

### Molecular Dynamics Simulations

The structures were minimized with the sander module each in two steps. Positional restraints (10 kcal mol^−1^ Å^−2^) were applied on the RNA molecule in the first step of minimization with 5,000 steps of steepest-descent algorithm and subsequently followed with the second round of minimization with 5,000 steps of conjugate-gradient algorithm and no restraints. Minimization was followed by an equilibration protocol first in constant volume with restraints on the RNA molecule (10 kcal mol^−1^ Å^−2^) and gradually increasing the temperature up to 300 K for several nanoseconds using the Langevin thermostat. The second round of equilibration was performed at constant pressure with constant temperature at 300 K and pressure coupling (Berendsen et al., 1984) of 1.0 ps^−1^ gradually removing the constraints on the solute. After minimization and equilibration, MD simulation under constant pressure (NPT) with a 2 fs time step was performed for each system with isotropic positional scaling. The reference pressure was set to 1 atm with a pressure relaxation time of 2 fs. SHAKE (Ryckaert et al., 1977) was turned on for constraining bonds involving hydrogen atoms. An atom-based long-range cutoff of 10.0 Å was used in the production runs. The reference temperature was set to 300 K. The Particle Mesh Ewald (PME) method was used to handle the electrostatics (Essmann et al., 1995) and the Langevin thermostat (Liu et al., 2016) was applied with a coupling constant *γ* = 1.0 ps^−1^ (Supplementary Table S1). Simulations were performed using the pmemd.CUDA implementation of AMBER 16. Total simulation time over 380 µs was invested to explore the conformational variability adopted by the internal loops as well as the bending propensity of these constructs consuming over 40 K GPU hours (see details below).

### Analyses

Base pair step parameters, groove widths as well as bending angles, and curvilinear helical axis, were measured using Curves^+^ (Lavery et al., 2009) and 3DNA (Lu and Olson, 2008). The first five terminal base pairs at each end were excluded from the calculations. We have used the refined value of groove widths calculated by 3DNA. In order to calculate the kink angles, 3DNA was used to calculate the normalized vector along the helical axis. Kink was consequently measured as the angle between two axes identified for each section of the kinked RNA (straight and bent). Cluster analyses were performed using the k-means algorithm implemented in the cpptraj module of AMBER16. Cpptraj was also used to calculate the average structures for each corresponding cluster. The number of target clusters was varied between 8 and 20 using all heavy atoms to capture different conformational ensembles experienced along the trajectory. In-house codes were utilized to investigate the structural details of individual RNA internal loops and to perform 2D population analyses. Each RNA model we studied has different repeat sizes (Table 1). Thus, while performing cluster analyses for each system, we combined all the internal loops each structure maintains except the first and last internal loops. Because 2×AUUCU, 2×CAG, and 2×CUG each have only two internal loops, both loops were included in the analyses. Symmetry observed in the RNA internal loops were included while performing the cluster analyses. Furthermore, only the first 54 µs MD trajectory of 2×AUUCU was included in the analyses because the RNA structure gets distorted dramatically afterward. Finally, no base pair step analyses were performed on 2×CUG because the MD trajectory displays a stable state where one of the end strands unfolds and forms a triple-stranded RNA structure, which does not allow for meaningful base pair step analyses. We discovered that bending in 20×CUG, 20×CAG, 15×CCUG, and 10×AUUCU are coordinated with the changes in the major groove widths (Mgw) adopting unique values varying between 10 Å and 18 Å depending on the sequence and the type of the internal loop (see details below).

## Results and Discussions

### AUUCU Repeats Display Both Bent and Kinked Structures

**FIGURE 1:**
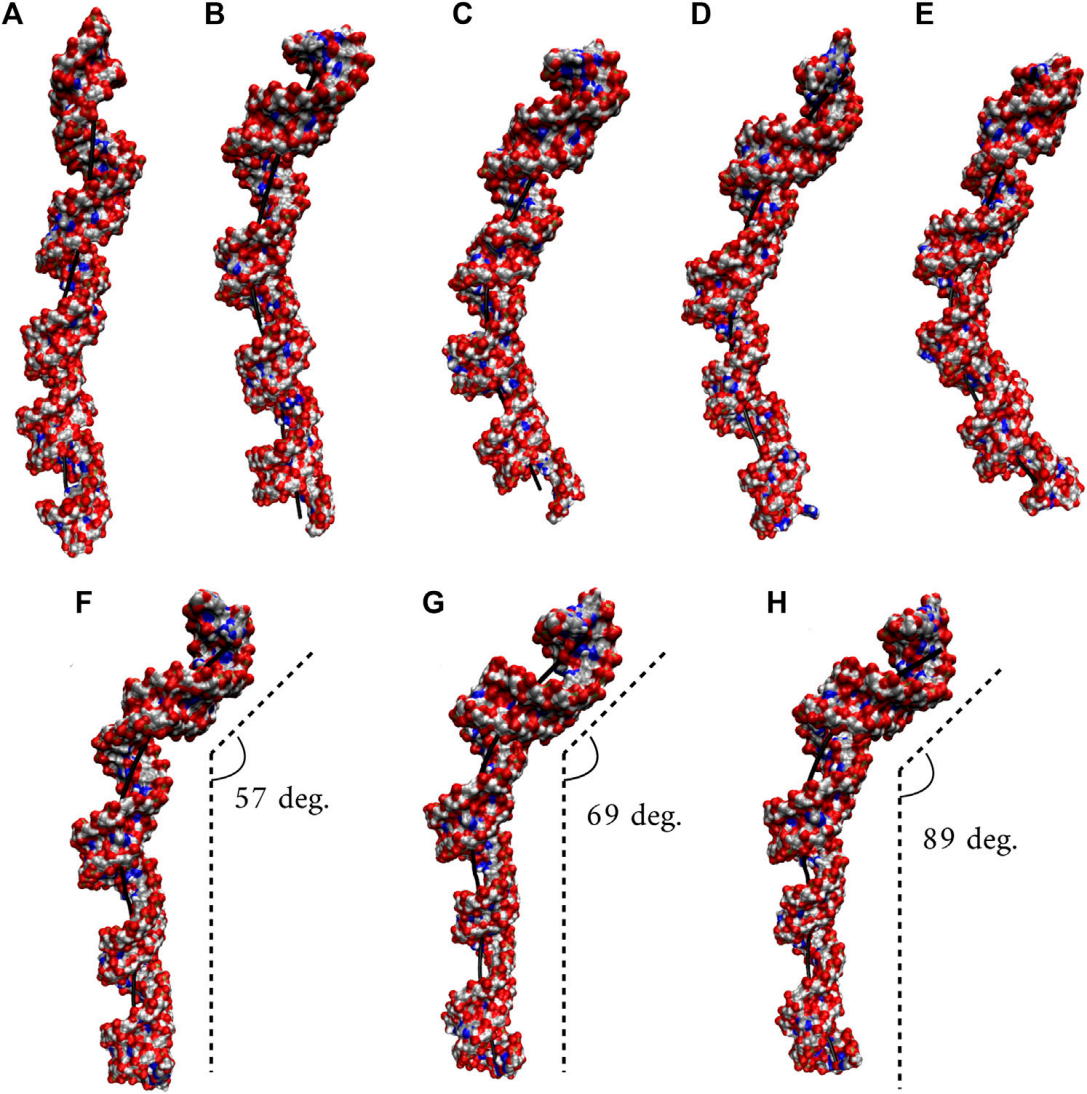
Bent **(A–E)** and kink **(F–H)** states observed in 10×AUUCU. Each structure in A–E represents different bent states displayed in Table 2. For example, structure A is a representative of the structures with a bending angle between 0° and 20°. No clustered state with bending angles between 20° and 30° is observed in 10×AUUCU (Table 2). The curvilinear helical axis is shown as a bold black line to emphasize the curvature in each case. A kinked structure does not have a continuous curvilinear axis such as those observed in **(A–E)**.

We utilized an RNA model, 10×AUUCU, and performed 2.9 µs MD simulation to investigate the global structural behavior of AUUCU repeats responsible for SCA10. The 10×AUUCU includes 10 copies of 3×3 UCU/UCU internal loop motifs, which makes it a realistic system to investigate AUUCU repeats. MD trajectory of 10×AUUCU exhibits structures having both bent (Figures 1A–E) and kink geometries (Figures 1F–H). When a continuous curvilinear axis is applicable to RNA helix, the term “bent” is used. The term “kink” refers to RNA structures when axial bent is observed in just one section. Cluster analyses of 10×AUUCU showed that over 70% of the populations are bent with bending angles >40° (Figures 1C–E and Table 2). Furthermore, more than 20% of the structures display extremely bent geometries with bending angles >60°, an extremity which is not observed in any other RNA system (Figure 1E). Another important conformational ensemble observed in 10×AUUCU are the structures having sharp kinks observed within two full RNA turns corresponding to ∼24 base pairs (Figures 1F–H). The presence of 3×3 UCU/UCU internal loops in 10×AUUCU creates distortions in the RNA backbone forming kink-like structures with kink angles ranging between 50° and 90°. Also, time evolution analysis of the bending angle along the trajectory showed the changes of the bending angle, settling down around an average value of ∼40° (Supplementary Figure S2).

**TABLE 2:** Bending angles calculated for average structures of clusters extracted from MD trajectories.

Bending Angle^a(°)^	10×AUUCU (%)	4×AUUC (%)	20×CUG (%)	10×CUG (%)	20×CAG (%)	10×CAG (%)	15×CCUG (%)	10×CCUG (%)
0–20	23.2	54.9	19.8	64.7	12.58	63.3	23.7	43
20–30	0	25.9	39.5	22.5	7.2	28.8	22.8	18.2
30–40	2.6	16.9	14.8	11.9	28.6	7.2	15.5	34.8
40–50	36.51	0	11	0	17.81	0	13.6	3.7
50–60	17	1.9	6.9	0	15.18	0	13	0
>60	20.3	0	7.69	0	18.07	0	11	0
Average Angle (°)	42.2	21.1	35.6	22.9	51.6	24.5	39.4	29.5

a Structures with bending values between 0° − 40°, 40° − 50°, and >50° are considered “moderately”, “strongly”, and “extremely” bent states, respectively.

Note that first and last five base pairs are excluded from the calculations.

While a perfect A-form RNA has a Mgw around 12 Å, analyses of the two most bent states with bending angles of 69° and 74° observed in 10×AUUCU display an increase in Mgw with an average value of 18 Å (Supplementary Figure S3). Furthermore, at certain points in the structure, collapse of Mgw is observed where Mgw is noticeably decreased to a value < ∼12 Å. Analyses of the clustered states of 10 ×AUUCU display that the total number of collapsed Mgw increase with the bending angle (Supplementary Figure S4). Results are in line with previous studies where one of the contributing factors for bending in RNA having AU-tracts was the collapse of Mgw. (Marin-Gonzalez et al., 2020).

### RNA CUG Repeats Exhibit Both Bent and Overextended Geometries

**FIGURE 2:**
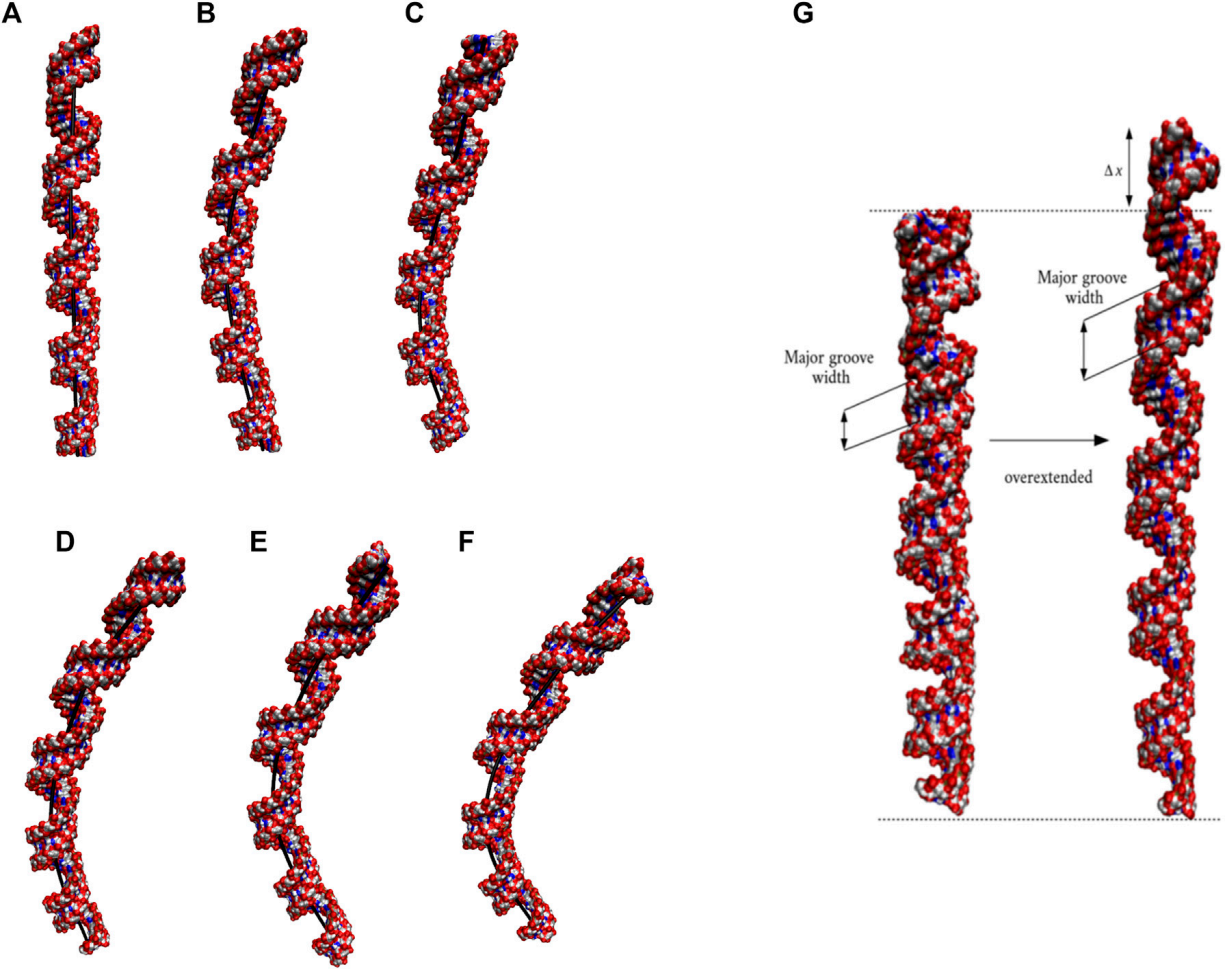
Bending **(A–F)** and over-extension **(G)** observed in 20×CUG. Each structure in **(A–F)** represents different bent states displayed in Table 2. The curvilinear helical axis is shown as a bold black line to emphasize the curvature in each case. While average rise value in relaxed structure is 2.8 Å, over-extended conformation has average rise value around 3.3 Å due to increase in major groove width.

The 20×CUG has 20 copies of CUG that makes it a more realistic model to study r(CUG)^exp^. We performed 2.6-µs-long MD simulation on this system to determine the bending properties of r(CUG)^exp^. To identify the bending, cluster analyses were conducted on the MD trajectory, average structures for each cluster were calculated, and structural analyses were performed on the average structures using the Curves^+^. Visual inspection of average structures, which correspond to states with different bending angles (Table 2), revealed a range of distinct bent conformations (Figures 2A–F), as well as overextended RNA geometries (Figure 2G and Supplementary Figure S5). Inspection of structural parameters showed that increase in the major groove widths and decrease in the average twist angles are fingerprints of overextended RNA conformations compared with the relaxed A-form. While the average twist angle calculated for the relaxed structure is 31°, this value drops to 28° in the overextended conformations. Furthermore, average helical rise increases from 2.8 Å observed in relaxed A-form RNA to 3.4 Å in the overextended conformations (Supplementary Table S2). The changes in twist and rise accompanied by the increase in the major groove width cause 20×CUG to display an overextended geometry (Figure 2G). The coupling of twisting and stretching in RNA has already been reported, where it was found that helical rise is inversely correlated with twist angle in double stranded RNA structures, meaning that overstretched RNA duplexes have under-twisted geometries. (Marin-Gonzalez et al., 2020). Although we observed a similar pattern, it should be noted that the presence of base pair mismatches in the internal loops of 20×CUG adds up complexity to the analysis. While overextension is one of the structural features observed in 20×CUG, bending is another important phenomenon we discovered. Measurement of bending angles of the average structures representing each cluster revealed that more than 80% of the total conformations have a curvature between 0° and 50° (Figures 2A–D). Furthermore, more than 14% of the population exhibit extreme bending with curvatures greater than 50° (Figures 2E, F and Table 2). Analysis of Mgw of these bent states reveals that the collapse of Mgw is again the main reason for the observed curvature (Supplementary Figure S6). Similar to the results observed in 10×AUUCU, as the total number of collapsed Mgw increases so does the bending angles in 20×CUG (Supplementary Figure S4).

### Extreme Bending Is Observed in CAG Repeats due to Bulky 1× 1 A/A Loops

**FIGURE 3:**
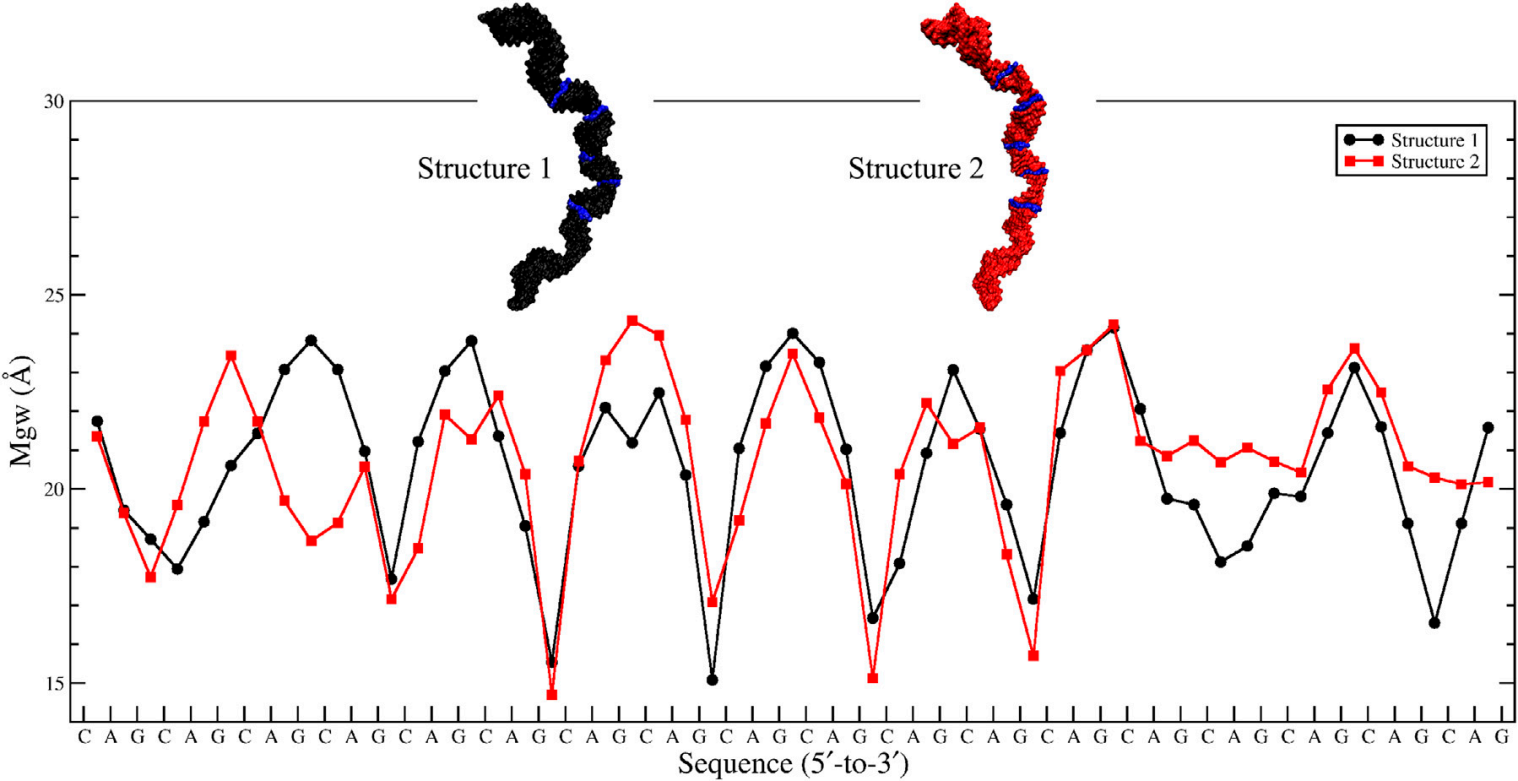
Major-groove width analyses of the two most bent clusters observed in 20×CAG. Changes in Mgw from an average value of ∼20 Å to ∼15 Å in a zigzag-like pattern, which create extremely bent conformations, are highlighted in blue in each structure.

Based on the 20×CUG results, similar bending behavior was expected in 20×CAG. Cluster analysis and consequently structural analysis along with measurements of the bending angle showed that this system underwent a more extreme bending regime with more than 30% of the observed bent structures showing a curvature >50° (Table 2 and Supplementary Figures S7A–F). Analysis of the bending angle, along the MD trajectory, also displayed more pronounced changes in 20×CAG compared with the 20×CUG (Supplementary Figure S2). Furthermore, this construct displayed overstretched geometries (Supplementary Figures S7G and S8) accompanied by drastic changes in the Mgw along with some moderate changes in the rise parameter, being 2.8 Å in A-form RNA and 3.4 Å in the overstretched forms (Supplementary Table S2). Due to the presence of more bulky residues in the 1×1 A/A loops compared with the CUG repeats, changes in the Mgw along the RNA construct are more pronounced resulting in more stretched and bent conformations (Supplementary Figure S7). Changes in the twist angle from an average value of 32° in a standard A-form RNA to 27° as well as increase in the Mgw are the contributing factors to over-stretching observed in 20×CAG. As a general pattern observed before, changes in Mgw in 20×CAG is creating the observed curvature (Figure 3). Similar to the results observed in 10×AUUCU and 20×CUG, the total number of collapsed Mgw in 20×CAG is pretty much associated with the bent geometries observed in the clustered states. For example, the extremely bent states observed in 20×CAG have over 30 collapsed Mgw values out of 63 in the RNA structure, which displays the connection of global bending behavior observed in 20×CAG with the collapse of the Mgw (Supplementary Figure S4). A noteworthy difference between 20×CAG and the other systems studied is the zigzag-like patterns observed in the Mgw values along RNA, where the collapse of Mgw at several points along 20×CAG causes the structure to transform to extremely bent states (Figure 3).

### Moderate Bending is Observed in CCUG Repeats

We utilized an RNA model, 15×CCUG, and performed 1.8 µs long MD simulation to investigate the global structural behavior of CCUG repeat expansions. The 15×CCUG includes 15 copies of 2×2 CU/UC internal loops, which makes it as realistic as possible to investigate CCUG repeats. Due to partial similarities between 15×CCUG and 10×AUUCU it was expected that they will display similar properties. Measurements of the bending angles of the average structures calculated for the clustered states show that 75% of the observed conformations have bending angles <50° (Table 2 and Supplementary Figures S9A–D), while the rest of the structures display extremely bent states with bending angles >50° (Table 2 and Supplementary Figures S9E–F). It was observed that while the presence of an extra U/U mismatch in 10×AUUCU creates far more instability than expected, where extremely bent and kinked states are created, bending properties in 15×CCUG were toned down to have moderate curvature, where no kink formation was observed (Supplementary Figures S9A–F). Similar to the other cases, overstretching was observed in this construct caused by increase in Mgw. In 15×CCUG, average Mgw is around 20 Å both in over-stretched and bent conformations (Supplementary Figure S9G). Similar to the other systems, the collapse of Mgw values is connected with bending (Supplementary Figure S10).

### Excessive Bending Observed in Expanded Repeats is an Interplay Between Intrinsic Bendability and Presence of Mismatch Pairs

In order to investigate the effect of mismatch pairs in the excessive bending regime observed in RNA expanded repeats, we prepared fully Watson-Crick (WC) base-paired RNA helixes for 20×CAG, 20×CUG, 15×CCUG, and 10×AUUCU and analyzed the changes of bending angle along the trajectory. It was observed that the average bending angles of these systems are 10°–20° less compared with the same systems with mismatch pairs (Supplementary Figure S11and Supplementary Table S3). This difference is especially noticeable in 20×CAG where the average bending angle for the expanded repeat is 20° more than the fully WC base-paired RNA construct.

### 3×3 UCU/UCU Internal Loops in AUUCU Repeats Display UU Pairs Forming 2 Hydrogen Bond States While CC Pairs Forming Dynamic States

**FIGURE 4:**
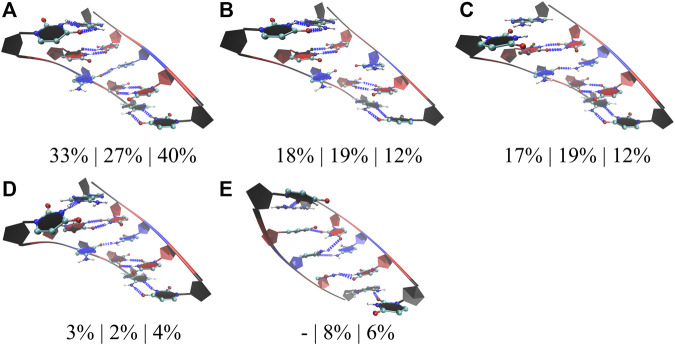
Cluster analyses performed on three model RNA systems, 2×AUUCU, 4×AUUCU, and 10×AUUCU, mimicking AUUCU repeat expansions. Panels **(A–E)** display stable states observed in the MD trajectories such as states with one **(A,C,D)**, zero **(B)**, and two hydrogen bond states **(E)** observed in 1×1 CC non-canonical base pairs. Residues colored in red, and blue represent uridine and cytidine, respectively, while closing base pairs are displayed in black and in new ribbon forms. Dashed blue lines represent the hydrogen bonds observed in the structures. Three percentages displayed under each structure represent how often the conformations are observed in the MD trajectories of the three model systems in the same order described above. Note that in **(C,D,E)**, one or both of the closing AU base pairs are distorted.

In the studies of RNA AUUCU repeats, three RNA constructs each having two, four, and ten copies of AUUCU loops were utilized (2×AUUCU, 4×AUUCU, and 10×AUUCU) (Table 1). Population distribution analyses performed on 10×AUUCU are as realistic as possible to represent the properties of 3×3 UCU internal loops observed in AUUCU repeat expansions compared with 2×AUUCU and 4×AUUCU. Nevertheless, 10×AUUCU has more than 50 base pairs, making it hard to run long MD simulations. While we run 90- and 26-µs-long MD simulations on 2×AUUCU and 4×AUUCU, respectively, only 2.9-µs-long MD simulation was run on 10×AUUCU. RNA AUUCU repeats are uridine-rich sequences with repeating 3×3 UCU internal loop motifs connected with 2×2 AU/UA Watson Crick base pairs. In anti-orientation, UU and CC pairs can form a maximum of two and one hydrogen bonds, respectively, which we have observed in the analyses. Cluster analyses revealed that the most populated state in all three model systems was when all the uridines in 3×3 UCU loop were forming UU pairs with two hydrogen bonds, while cytidines were forming CC pairs with one hydrogen bond that was observed 33%, 27%, and 40% in 2×AUUCU, 4×AUUCU, and 10×AUUCU, respectively (Figure 4A). Moreover, a stable state, where CC is in 0 hydrogen-bond state and both UU are in 2 hydrogen-bond states, was observed 18%, 19%, and 12% in 2×AUUCU, 4×AUUCU, and 10×AUUCU, respectively (Figure 4B). It was observed that CC pairs are dynamic and can form 0 and 1 hydrogen bond, while UU pairs stay in two hydrogen-bond states most of the time in RNA AUUCU repeats. Cluster analyses further discovered states, which were similar to the global minimum (Figure 4A) but with one of the closing AU base pairs in a distorted state having 0 or 1 hydrogen bond (Figures 4C, D). In 4×AUUCU and 10×AUUCU, a stable state, where both closing AU base pairs are distorted, is observed for 8% and 6%, respectively. AU base pairs lacks one hydrogen bond compared with GC base pairs and, thus, are more prone to distortions and bending compared with GC pairs. Recently, it has been shown that insertion of AU tracts within a purely GC rich sequence introduced stress causing bending. (Marin-Gonzalez et al., 2020). Results imply that as the AUUCU repeat size increases unique conformations such as distorted closing AU pairs are observed, which can play crucial roles in AUUCU repeats interacting with hnRNP K.

### 2 × 2 CU/UC Internal Loops in CCUG Repeat Expansions Display CU Pairs Forming 0, 1, and 2 Hydrogen Bond States

**FIGURE 5:**
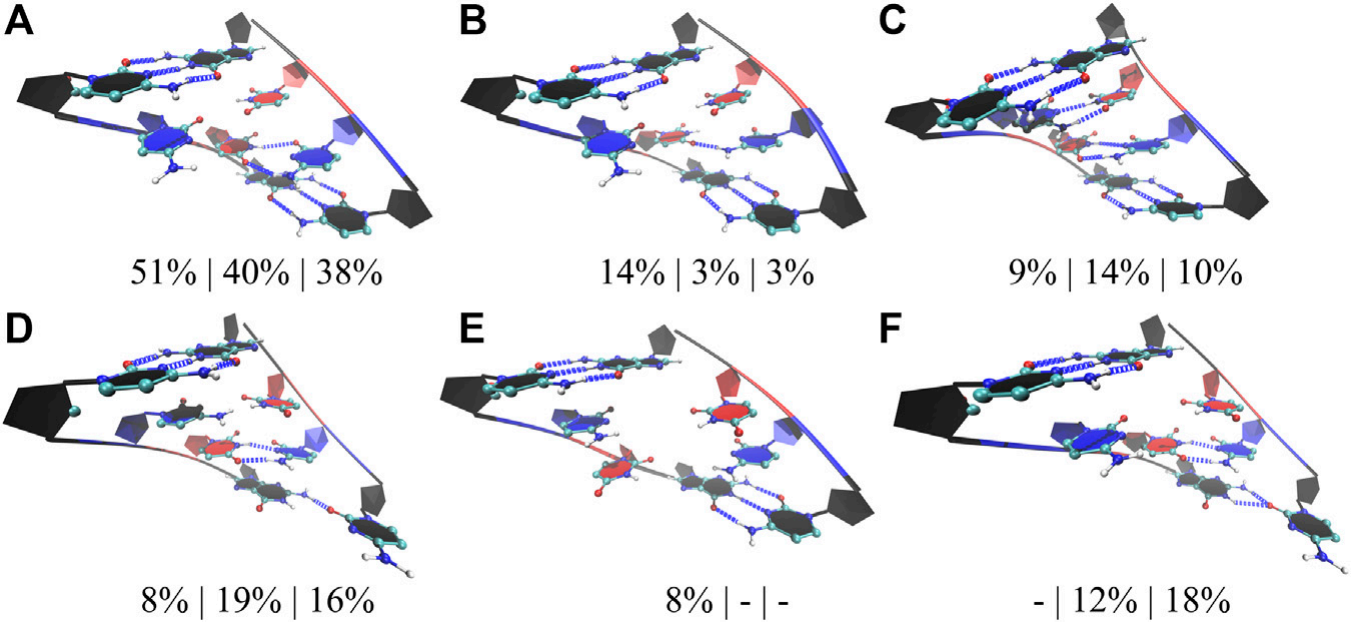
Cluster analyses performed on three model RNA systems, 4×CCUG, 10×CCUG, and 15×CCUG, mimicking CCUG repeat expansions. Panels **(A–F)** display stable states observed in the MD trajectories such as one of the CU base pairs in two hydrogen bond state **(A,D,F)**, one of the CU base pairs in a single hydrogen bond state **(B)**, both of the CU base pairs in two hydrogen bond states **(C)**, and both of the CU base pairs forming no hydrogen bond states **(E)**. Note that in d and f, one of the closing GC base pairs are distorted forming a single hydrogen bond. Residues colored in red, and blue represent uridine and cytidine, respectively, while closing base pairs are displayed in black and in new ribbon forms. Dashed blue lines represent the hydrogen bonds observed in the structures. Three percentages displayed under each structure represent how often the structures are observed in the MD trajectories of the three model systems in the same order described above.

We followed a similar approach in the studies of RNA CCUG repeats, where three models with 4, 10, and 15 copies of CCUG loops were investigated (4×CCUG, 10×CCUG, and 15×CCUG) (Table 1). Similar to 10×AUUCU, 10×CCUG and 15×CCUG are realistic models to represent properties of 2×2 CU internal loop motifs observed in RNA CCUG repeat expansions, but due to the system size MD simulation times were limited (Table 1). RNA CCUG repeats are cytidine-rich sequences with repeating 2×2 CU internal loop motifs connected with 2×2 GC/CG Watson– Crick base pairs. In anti-orientations, CU pairs can form a maximum of two hydrogen bonds. Indeed, cluster analyses performed on 4×CCUG, 10×CCUG, and 15×CCUG display that 51%, 40%, and 38%, respectively, of structures prefer a stable state where one of the CU pairs is forming a 2 hydrogen-bond state while the other one forming a 0 hydrogen bond state (Figure 5A). Another stable state, where one of the CU is in a single hydrogen-bond state while the other one is in zero-hydrogen-bond state, is observed 14%, 3%, and 3% in 4×CCUG, 10×CCUG, and 15×CCUG, respectively (Figure 5B). The case where both CU pairs have two-hydrogen-bond state is observed between 9% and 14% in the model systems, which is counterintuitive as one would expect this conformation to be the most preferred state (Figure 5C). Cytidine and uridine are pyrimidine residues, which are six-membered heterocycles, while guanosine and adenosine are purines, which are nine-membered double-ring systems. The natural form of RNA is A-form, which is created by Watson–Crick AU, GU, and GC base pairs. The shape of a purine-pyrimidine base pair, such as AU, GU, and GC, cannot be captured by a pyrimidine-pyrimidine base pair, such as the case when CU pairs have two hydrogen-bond states. When two of the CU pairs in CCUG motif would prefer the two-hydrogen-bond state as displayed in Figure 5C, there will be stress imposed on the RNA backbone to distort the natural A-form orientation, which is one of the reasons why this structural motif is not the dominant state observed in RNA CCUG repeats. It is possible that this stress causes one of the CU pairs to transform to 0 hydrogen-bond state as observed in Figure 5A, or causes either both of the CU pairs to form zero hydrogen bond states or one of the terminal GC base pairs to lose its hydrogen bonds as observed in Figures 5D–F. Both CU pairs in 0 hydrogen-bond state was also observed in 4×CCUG by 8% (Figure 5E). Furthermore, compared with 4×CCUG, 10×CCUG and 15×CCUG have more stable structures with one of the closing GC base pairs in distorted form (Figures 5D,F). For example, the clustered state displayed in Figure 5D is observed 8%, 19%, and 16% while the clustered state displayed in Figure 5F is observed 0%, 12%, and 18% in 4×CCUG, 10×CCUG, and 15×CCUG, respectively. Similar to the results observed in AUUCU, as the repeat size increases in CCUG repeat expansions, stable states with distorted GC closing base pairs (Figures 5D,F) start to show up in RNA structure, which can have crucial roles in CCUG repeats interacting with MBNL1.

### 1×1 AA and UU Internal Loops, Respectively, in CAG and CUG Repeats Display Dynamic States With 0, 1, and 2 Hydrogen Bonds

**FIGURE 6:**
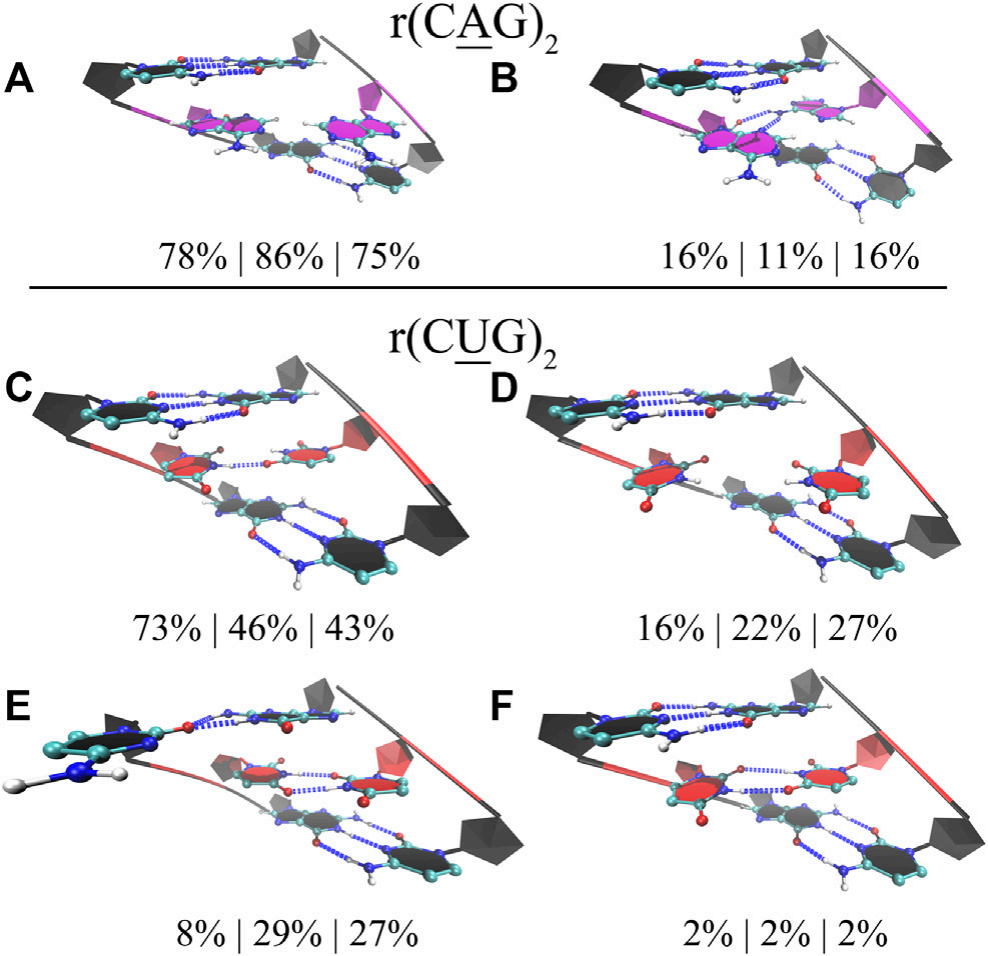
Cluster analyses performed on model RNA systems mimicking CAG and CUG repeats. Panels a to f display stable states observed in the MD trajectories such as zero **(A)** and one hydrogen bond **(B)** states observed in 1×1 AA internal loops, and one **(C)**, zero **(D)**, and two hydrogen bonds **(E,F)** states observed in 1×1 UU internal loops. Residues colored in red, blue, and magenta represent uridine, cytidine, and adenosine, respectively, while closing base pairs are displayed in black and in new ribbons form. Dashed blue lines represent the hydrogen bonds observed in the structures. Three model systems were utilized to study each RNA repeat, where 2×CAG, 10×CAG, and 20×CAG were used to investigate CAG repeats and 2×CUG, 10×CUG, and 20×CUG were used to investigate CUG repeats. Three percentages displayed under each structure represent how often the structures were observed in the MD trajectories of the three model systems in the same order described above.

Finally, for completeness, we also studied the properties of 1×1 AA and UU internal loops observed in RNA CAG and CUG repeat expansions. Again, we used three different model systems in each case having 2, 10, and 20 copies of CAG (2×CAG, 10×CAG, and 20×CAG) and CUG loops (2×CUG, 10×CUG, and 20×CUG) (Table 1). Previous studies found that 1×1 AA internal loops in CAG repeats preferred both zero- and one-hydrogen-bond states, while 1 × 1 UU internal loops in CUG repeats preferred zero-, one-, and two-hydrogen-bond states (Yildirim et al., 2013; Yildirim et al., 2015; Chen et al., 2017). We observed similar results in 2×CAG, 10×CAG, and 20×CAG, where 0 hydrogen bond states were observed 78%, 86%, and 75%, respectively, while one-hydrogen-bond states were observed 16%, 11%, and 16%, respectively (Figures 6A,B). In one-hydrogen-bond state, amino group of one of adenosine is in close contact with N3 and 2′–OH group of the other loop adenosine residue that stabilizes the conformation (Figure 6B). Furthermore, cluster analyses of 2×CUG, 10×CUG, and 20×CUG showed that 1 hydrogen-bond state of UU in RNA CUG repeats was preferred 73%, 46%, and 43%, respectively (Figure 6C), while 0 hydrogen-bond state was preferred 16%, 22%, and 27%, respectively (Figure 6D). Similar to the previous studies (Yildirim et al., 2015; Chen et al., 2017), one of the closing GC base pairs in RNA CUG repeats could get distorted, which was observed 8%, 29%, and 27% in 2×CUG, 10×CUG, and 20×CUG, respectively (Figure 6E). Analogous to the results of AUUCU and CCUG repeats, as the repeat size increases in RNA CUG repeat expansions, stable states with distorted GC closing base pairs start to appear more, which could be important in how MBNL1 would target RNA CUG repeats. Moreover, as described above, in anti-orientation, UU pairs can have a maximum of 2 hydrogen-bonds, which is also observed in model RNA CUG repeats but only 2% of times in all the model systems (Figure 6F). This is again probably due to the size of UU pairs being smaller than Watson-Crick base pairs causing stress on the RNA backbone and transforming into an unfavorable backbone conformation. In order to stabilize the two-hydrogen-bond UU state, one of the closing GC base pair gets distorted as seen in Figure 6E. As a result, UU pairs in RNA CUG repeats tend to stay away from two-hydrogen-bond states (Figure 6F), and sample mainly one- and zero-hydrogen-bond states (Figures 6C,D).

### Distortions Observed in Closing Base Pairs in RNA Repeats Is Coordinated With the Extreme Bending Observed in 10×AUUCU, 15×CCUG, 20×CAG, 20×CUG

**FIGURE 7:**
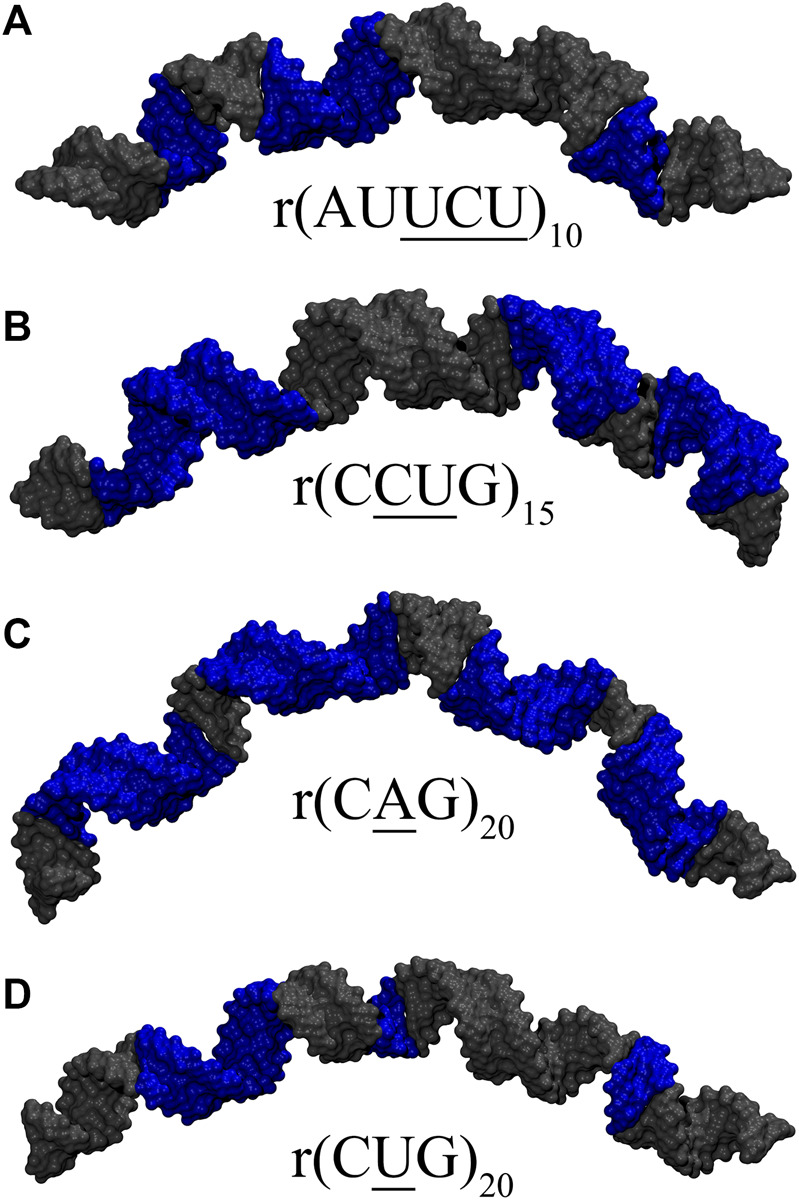
Loop conformations observed in representative clusters having extreme bending in **(A)** 10×AUUCU, **(B)** 15×CCUG, **(C)** 20×CAG, and **(D)** 20×CUG. Structures highlighted in Supplementary Table S4 are displayed in the figure. Blue colored regions in **(A,B)** are RNA loop conformations having distorted closing GC or AU base pairs (Figures 4C–E, 5D,F), while in **(C,D)**, they represent weakest RNA loop conformations (Figures 6A,D,E). See Supplementary Figure S12 for details.

As discussed above, RNA AUUCU, CCUG, CAG, and CUG repeats have tendencies to bend dramatically with the increase in repeat size. The native form of RNA is A-form, which is a linear structure stabilized by hydrogen bonds formed by Watson-Crick GC, AU, and GU base pairs. Defects in RNA structure such as formation of internal loops can distort the global RNA structure and form states, which are bent or display kink-like structures. RNA repeat expansions have regularly spaced internal loops such as 3×3 UCU/UCU in AUUCU repeats, 2×2 CU/UC in CCUG repeats, 1×1 A/A in CAG repeats, and 1×1 U/U in CUG repeats, which can have global structural implications due to the dynamic behavior of the internal loops (Figures 4–6). For example, 3×3 UCU/UCU in AUUCU displays states where one or both of the closing AU base pairs are distorted probably due to formation of three pyrimidine-pyrimidine non-canonical base pairs, which place stress on the RNA backbone and cause AU base pairs to lose hydrogen bonds (Figures 4C–E). These distorted states are observed in structures displaying extreme bending in 10×AUUCU (Figure 7A, Supplementary Figure S12A, and Supplementary Table S4). Similar results were noticed in 15×CCUG, too. Cluster analyses performed on 2× 2 CU/UC internal loops of 15×CCUG display two stable states having one of the closing GC base pairs in a single hydrogen bond state for over 16% of time (Figures 5D, F). These two distorted states are observed in bent conformations of 15×CCUG (Figure 7B, Supplementary Figure S12B, and Supplementary Table S4). Finally, investigation of 20×CAG and 20×CUG show similar results, but a different mechanism for extreme bending phenomenon. 1×1 AA internal loops in CAG repeats prefer zero and one hydrogen bond states, where in the extreme bent cases 1×1 AA internal loops are mostly in zero hydrogen bond states (Figure 7C, Supplementary Figure S12C, and Supplementary Table S4). In contrast to UC, UU, and CC pairs, AA is a purine-purine pair distorting the groove widths, which is the main reason why 20×CAG forms extremely bent states (Figure 7C). In 20×CUG, distorted closing GC base are observed 27% of the time in the MD simulations (Figure 6E) that is one of the reasons why this system displays extreme bending (Figure 7D, Supplementary Figure S12D, and Supplementary Table S4). Such bent states in RNA repeat expansions are important as they might have important roles in binding mechanism with proteins such as hnRNP K and MBNL1.

### Extreme Bending is a Length Dependent Phenomenon

**FIGURE 8:**
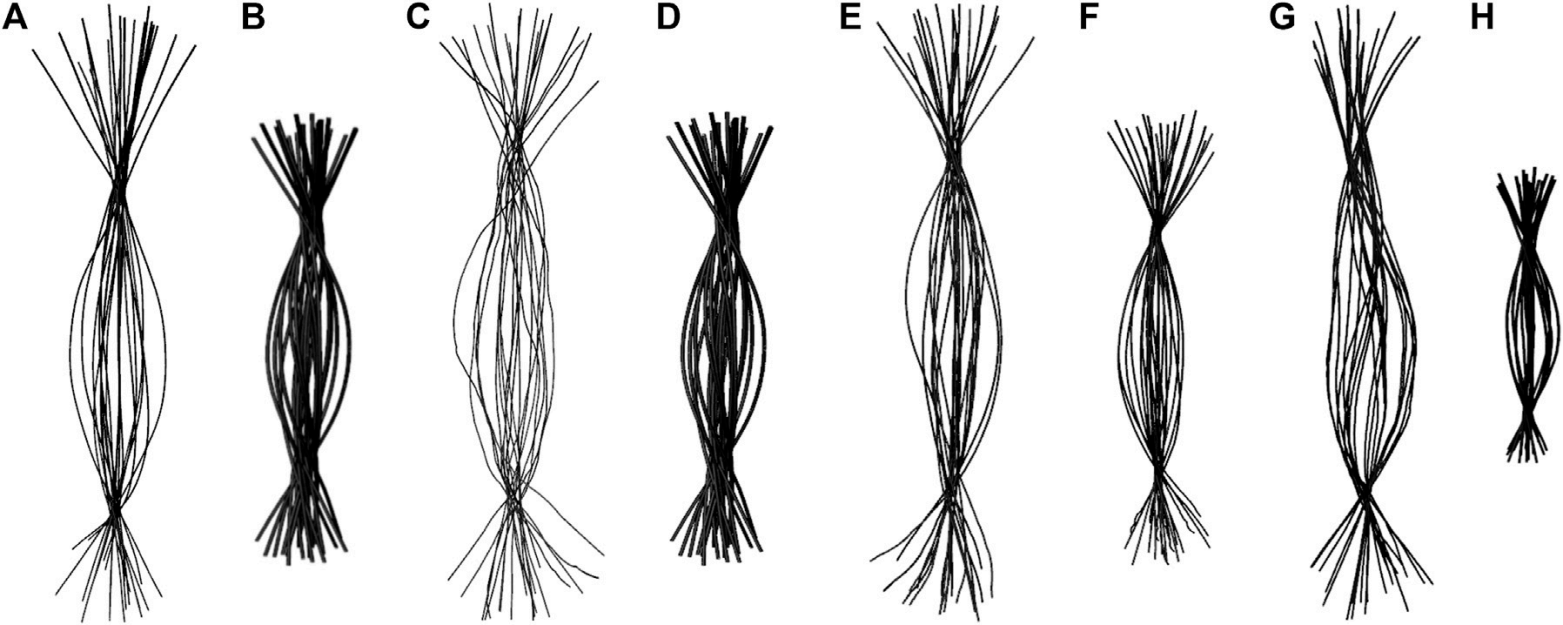
Overlay of the curvilinear helical axis in 20×CUG **(A)**, 10×CUG **(B)**, 20×CAG **(C)**, 10×CAG **(D)**, 15×CCUG **(E)**, 10×CCUG **(F)**, 10×AUUCU **(G)**, and 4×AUUCU **(H)**. Curvilinear axis was measured for each average structure of the 20 clusters, and overlayed to provide a visual inspection of the degree of bending observed in each system.

To determine if RNA bending is a length dependent phenomenon, we compared 20×CUG, 20×CAG, 15×CCUG, and 10×AUUCU to 10×CUG, 10×CAG, 10×CCUG, and 4×AUUCU, respectively. By applying the same methodology described above, we discovered that bending in 10×CAG and 10×CUG is limited to bending angles <40° (Table 2 and Supplementary Figure S2). The same inclination holds true for 10×CCUG, but with only 3.7% of the structures having bending angles >40° (Table 2 and Supplementary Figure S2). No extreme bending is observed in any of these systems. Results might provide explanations for why higher order structures are observed in expanded RNA repeats such as minimum number of repeats required for extreme bending. The relationship between the system size and the magnitude of bending is depicted in the overlap of the curvilinear helical axis (Figure 8). Although 10×CUG display different bent states, no states were observed having extreme bending (Figures 8A,B). Furthermore, the extremely bent geometries observed in 20×CAG are not observed in 10×CAG (Figures 8C,D), which highlights the importance of repeat size in bending phenomenon detected in RNA repeat expansions. The bending behavior observed in RNA CCUG is to a greater extent like the bending behavior identified for RNA CUG repeats (Figures 8E, F). Although the bending regime in 15×CCUG and 10×CCUG is almost the same, no extreme bending is observed in 10×CCUG (Table 2 and Supplementary Figure S2). In 10×AUUCU, majority of bent states are categorized as extremely bent, while in 4×AUUCU, more than 80% of bent conformations experience a bending less than 30° (Table 2 and Figures 8G,H).

### Local Distortion Originating at the Internal Loops Are Transferred to Global RNA Structure via Changes Resulting in Base Pair Steps

**FIGURE 9:**
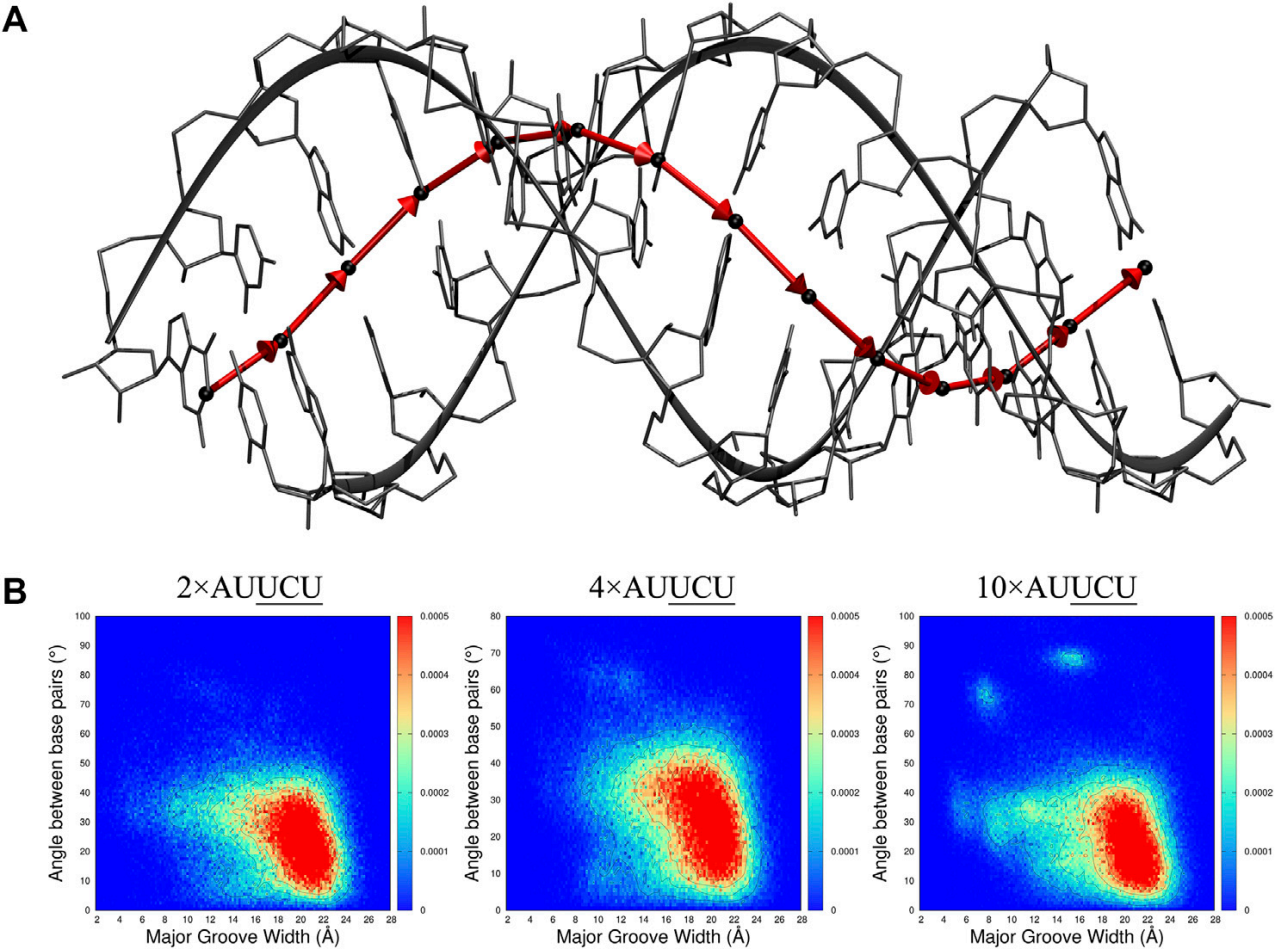
2D distribution analyses of major groove width (Mgw) and a reaction coordinate **(B)** representing angular change (θ) of base pair step orientation **(A)**. In the model system displayed in A, center of mass (COM) of each base pair is represented with black spheres, and vectors connecting the COMs of consecutive base pairs are displayed with red arrows. The angular change of the vectors is used as a reaction coordinate to investigate bending observed in RNA repeat expansions. In B, 2D (Mgw,θ) distribution analyses of 2×AUUCU, 4×AUUCU, and 10×AUUCU are shown. Note that as the repeat size increases, unique (Mgw,θ) regions are observed in the plots such as (Mgw = 7 Å, θ = 70°) and (Mgw = 16 Å, θ = 90°).

r(AUUCU)^exp^, r(CUCG)^exp^, r(CAG)^exp^, and r(CUG)^exp^ have repeating units with unique internal loops. At atomic level, electrostatics, stacking, and hydrogen bonding interactions are directing the structural preferences of bases in each internal loop, which will create local distortions. The combined effect of all these distortions generates the bending phenomenon discussed above. We already discovered that collapse of Mgw is coordinated with bending phenomenon. Thus, we investigated the connection of Mgw with minor groove width (mgw) and base pair step parameters (tilt, roll, twist, shift, slide, and rise) (Supplementary Figure S13) in order to determine the mechanism linking local distortions to global bending phenomenon. Furthermore, we calculated the angular change of base pair steps to find a link with Mgw (Figure 9A). One thing to note here is that the base pair step parameters are designed mostly to study regular RNA duplexes having Watson–Crick GC, AU, and GU base pairs. When dynamic internal loops are present in the system, results of base pair step parameters might be challenging to interpret. Nevertheless, we calculated the 2D population distributions for the model RNA constructs to determine any connection with the repeat size. We do not include the results of 4×CCUG because MD trajectory of this system exhibit one of the end strands unfolding and forming a triple-stranded RNA structure, which does not provide any useful base pair step data. Furthermore, the bending mechanism in each RNA repeat expansion is not guaranteed to be the same as local distortions in 3×3 UCU/UCU, 2×2 CU/UC, 1×1 A/A, and 1×1 U/U are different as we discuss below.

The 2D (Mgw, θ) profiles, where Mgw and θ stand for major groove width and angular change of base pair step orientation (Figure 9A), respectively, were calculated for 2×AUUCU, 4×AUUCU, and 10×AUUCU (Figure 9B). Results display that, compared with 2×AUUCU, new populations are emerging around (Mgw = 12Å, *θ* = 60°) in 4×AUUCU, and around (Mgw = 11Å, *θ* = 35°) (Mgw = 7Å, *θ* = 70°) and (Mgw = 16Å, *θ* = 90°) in 10×AUUCU(Figure 9B). No genuine differences were observed when results of base pairs steps were compared with these three RNA constructs (Supplementary Figures S14, S15).

Comparison of 2D (Mgw,mgw), (Mgw,twist), and (Mgw,shift) profiles calculated for 10×CCUG and 15×CCUG display slight differences (Supplementary Figures S16–S18). For example, the (Mgw = 23Å,mgw = 13Å) (Mgw = 20Å,twist = 30°) (Mgw = 24Å,twist = 20°) (Mgw = 23Å,shift = -3Å) and (Mgw = 23Å,shift = 3Å) regions are more emphasized in 15×CCUG compared with 10×CCUG (Supplementary Figures S16–S18).

The base pair step data of RNA CAG and CUG models are particularly interesting because the 1×1 AA and 1×1 UU pairs in each RNA construct are well defined, which allows base pair step data to be more physical compared with AUUCU and CCUG. For example, analyses display that angular change as well as twist and slide are all important in analyzing properties of RNA CAG repeats (Supplementary Figures S19–S21). Compared with 2×CAG, 10×CAG, and 20×CAG have new populations emerging around (Mgw = 19 Å, *θ* = 35°) (Mgw = 17 Å, twist = 35°) (Mgw = 19 Å, twist = 65°) (Mgw = 20 Å, slide = −1.5 Å), and (Mgw = 17 Å, slide = −2.5 Å) (Supplementary Figures S19–S21). Increase in θ value implies bending. Some of the 2D distributions calculated for 10×CAG and 20×CAG have differences such as the pronounced states in 2D (Mgw,twist) profiles (Supplementary Figure S20. Furthermore, analyses of the RNA CUG constructs display the emergence of new populations around (Mgw = 24Å, mgw = 13 Å) (Mgw = 24 Å, *θ* = 15°), and (Mgw = 24 Å, *θ* = 30°) in 10×CUG and 20×CUG compared with 2×CUG (Supplementary Figures S22). As before, increase in θ value implies bending, which is observed in 10×CUG and 20×CUG. Moreover, other new populations emerge in 10×CUG and 20×CUG such as (Mgw = 24 Å, twist = 0°) (Mgw = 17 Å, twist = 45°) (Mgw = 21 Å, twist = 40°) (Mgw = 23 Å, slide = −3 Å), and (Mgw = 23 Å, slide = 3 Å) (Supplementary Figures S23–S24). Results imply unique links between Mgw and mgw, twist, slide, and shift as well as angular change of base pair steps, which are associated with global structural changes observed in r(AUUCU)^exp^, r(CUCG)^exp^, r(CAG)^exp^, and r(CUG)^exp^.

## Conclusion

Bending in nucleic acids, especially in DNA having A-tracts (Hizver et al., 2001), is a well-known phenomenon with important functional implications in gene regulation (Dickerson, 1998; Haran and Mohanty, 2009). Despite the fact that RNA molecules have numerous biologically important functions in cell, there is a noticeable scarcity of studies concerning the global RNA structural deformations, alone or in complex with proteins. For example, RNA bulges have been known for a while in induced bending in RNA double helices (Luebke and Tinoco, 1996), which provides an interaction site to proteins such as interferon-induced protein kinase, PKR (Zheng and Bevilacqua, 2000). It was shown that bent RNA adenosine bulges (A-bulge), after interacting with proteins, are straightened up providing an important insight on how proteins are able to recognize bent RNA structures and modulate their conformational properties (Zacharias and Hagerman, 1995; Zheng and Bevilacqua, 2000). Motivated with the growing interest in the underlying role of RNA in several neuromuscular diseases and the possibility of targeting RNA for therapeutic purposes, we investigated the local as well as global structural behavior of several RNA repeats, as the local deformations can translate into global structural changes having important implications in RNA–protein interactions. RNA AUUCU, CCUG, CAG, and CUG repeat expansions have repetitive 3×3 UCU/UCU, 2×2 CU/UC, 1×1 A/A, and 1×1 U/U internal loops, respectively, which are dynamic. We found that these RNA repeats have strong bending propensities due to the local structural changes observed in their repetitive internal loop motifs, which collapse the major groove width at different locations in RNA causing global structural change called bending phenomenon. Furthermore, we discovered that there is a link between the magnitude of bending and the system size as well as the RNA internal loop motifs in these RNA repeat expansions, where extreme bending is observed in sequentially long RNA repeats.

In summary, we utilized model RNA systems having biologically relevant repeat sizes to provide a glimpse on the behavior of RNA repeats both at global and local levels. Presence of multiple pyrimidine–pyrimidine mismatches in internal loops was found to impose structural stress in RNA resulting in bent or kinked conformations. While RNA CCUG and AUUCU repeats are alike, we found that latter system exhibited extreme bending regimes as well as kinked conformations. Furthermore, studies on RNA CAG revealed the role of bulky 1×1 A/A mismatches in creating extreme curvatures in RNA structure. We also studied the effect of RNA length on its global behavior in these repeat expansions. We found that extreme bending regimes are only observed in long systems, although bending was also observed in small systems. Analysis of structural parameters revealed that changes in major groove width were the general outcome for observed bent states in all the studied systems. Changes in major groove width are due to weak pairings observed in RNA loop motifs, which can either distort the closing base pairs or the loop structures to provide enough energetics to create local distortions in multiple locations along the RNA resulting bent geometries. Finally, all the model RNA repeats displayed overextension, which is attributed to widening of the major groove widths as well as undertwisting phenomenon. The combined results can provide an understanding on how proteins would interact with RNA repeat expansions. Furthermore, results might be utilized in structure-based drug designs specifically targeting RNA repeat expansions.

## Data Availability

The raw data supporting the conclusion of this article will be made available by the authors, without undue reservation.
