# Supplementary material for: Computational Investigation of Bending Properties of RNA AUUCU, CCUG, CAG, and CUG Repeat Expansions Associated With Neuromuscular Disorders
Source: Front Mol Biosci. 2022 Apr 11;9:830161. doi: 10.3389/fmolb.2022.830161 (PMC9037632; doi:10.3389/fmolb.2022.830161)
Supplement: Supplementary file 1 [file DataSheet1.docx]

Supporting Information

Computational Investigation of Bending Properties of RNA AUUCU, CCUG, CAG and CUG Repeat Expansions Associated with Neuromuscular Disorders

Amirhossein Taghavi^1,2^ and Ilyas Yildirim^1,2*^

^1^ Department of Chemistry and Biochemistry, Florida Atlantic University, Jupiter, FL 33458 USA

^2^ Department of Chemistry, The Scripps Research Institute, Jupiter, FL, 33458 USA

ataghavi@scripps.edu, iyildirim@fau.edu

| **Table of Contents** | | |
| --- | --- | --- |
| **Table S1** | Sample MD input file | **p. S4** |
| **Table S2** | Helical rise of average structures calculated for 20×CUG and 20×CAG | **p. S4** |
| **Table S3** | Average bending angle measured for fully WC double helixes. | **p. S4** |
| **Table S4** | Loop conformations observed in the average structures of the twenty clusters | **p. S5** |
| **Figure S1** | Secondary structure of extended repeats. | **p. S6** |
| **Figure S2** | Bending angle as a function of time calculated for 20×CUG, 20×CAG, 10×CAG, 10×CUG, 15×CCUG, 10×CCUG and 10×AUUCU | **p. S7** |
| **Figure S3** | Mgw analyses performed on the two most bent states observed in 10×AUUCU | **p. S8** |
| **Figure S4** | Average bending angle vs average number of collapsed Mgw observed in the clustered states of 10×AUUCU, 20×CUG, and 20×CAG | **p. S9** |
| **Figure S5** | Average structures of overstretched conformations observed in 20×CUG | **p. S10** |
| **Figure S6** | Mgw analyses of the two most bent states observed in 20×CUG | **p. S11** |
| **Figure S7** | Bending and over-extension observed in 20×CAG | **p. S12** |
| **Figure S8** | Average structures of overstretched conformations observed in 20×CAG | **p. S13** |
| **Figure S9** | Bending and over-extension observed in 15×CCUG | **p. S14** |
| **Figure S10** | Mgw analyses of the two most bent states observed in 15×CCUG | **p. S15** |
| **Figure S11** | Bending angle (**°**) as a function of time calculated for fully Watson-Crick base paired RNA constructs | **p. S16** |
| **Figure S12** | Loop conformations observed in extremely bent states of 10×AUUCU, 15×CCUG, 20×CAG, and 20×CUG | **p. S17** |
| **Figure S13** | Schematic representation of base-pair step parameters | **p. S18** |
| **Figure S14** | 2D distribution analyses performed on 2×AUUCU, 4×AUUCU, and 10×AUUCU (Mgw vs. tilt/roll/twist) | **p. S19** |
| **Figure S15** | 2D distribution analyses performed on 2×AUUCU, 4×AUUCU, and 10×AUUCU (Mgw vs. shift/slide/rise) | **p. S20** |
| **Figure S16** | 2D distribution analyses performed on 10×CCUG and 15×CCUG (Mgw vs. θ/mgw) | **p. S21** |
| **Figure S17** | 2D distribution analyses performed on 10×CCUG and 15×CCUG (Mgw vs. tilt/roll/twist) | **p. S22** |
| **Figure S18** | 2D distribution analyses performed on 10×CCUG and 15×CCUG (Mgw vs. shift/slide/rise) | **p. S23** |
| **Figure S19** | 2D distribution analyses performed on 2×CAG, 10×CAG, and 20×CAG (Mgw vs. θ/mgw) | **p. S24** |
| **Figure S20** | 2D distribution analyses performed on 2×CAG, 10×CAG, and 20×CAG (Mgw vs. tilt/roll/twist) | **p. S25** |
| **Figure S21** | 2D distribution analyses performed on 2×CAG, 10×CAG, and 20×CAG (Mgw vs. shift/slide/rise) | **p. S26** |
| **Figure S22** | 2D distribution analyses performed on 2×CUG, 10×CUG, and 20×CUG  (Mgw vs. θ/mgw) | **p. S27** |
| **Figure S23** | 2D distribution analyses performed on 2×CUG, 10×CUG, and 20×CUG (Mgw vs. tilt/roll/twist) | **p. S28** |
| **Figure S24** | 2D distribution analyses performed on 2×CUG, 10×CUG, and 20×CUG  (Mgw vs. shift/slide/rise) | **p. S29** |

**Table S1.** Sample input file used in MD simulations.

&cntrl

imin=0,

ntpr=25000,ntwr=100000,ntwx=25000,

ntb=2, cut=10.0, ntc=2,

ntt=3, temp0=300.0, tempi=300.0,

ntf=2, irest=1, ntx=5,

ntp=1, barostat=1, taup=2.0,

pres0 = 1.0,

gamma_ln=1.0, nstlim=50000000, dt=0.002, ntxo=2,

ig=-1,

ntr=0,

ntxo=2,

iwrap=0,

ioutfm=1

/

**Table S2.** Helical rise of average structures calculated for clusters representing overextended states. Average structures of the overstretched conformations are depicted in Figures S5 and S8.

|  | Helical rise (Å) | |
| --- | --- | --- |
| Conformations | 20×CUG | 20×CAG |
| A-RNA  Over Str. 1 | 2.81  3.28 | 2.81  3.43 |
| Over Str. 2 | 3.30 | 3.46 |
| Over Str. 3 | 3.31 | - |

**Table S3.** Average bending angle measured for fully WC double helixes.

| Model system (fully WC double helix) | Average bending angle (°) |
| --- | --- |
| r(CAG)_20_ | 33.09 |
| r(CUG)_20_ | 25.55 |
| r(AUUCU)_10_ | 32.22 |
| R(CCUG)_15_ | 22.04 |

**Table S4.** Loop conformations observed in the average structures of the twenty clusters extracted from the MD trajectories of 10×AUUCU, 15×CCUG, 20×CAG, and 20×CUG. Due to their dynamic nature, the first and last loops in each structure are not included in the analyses. Loop states displayed in Figures 4-6 are used to represent each loop in each structure. X denotes loop conformations, which cannot be represented by one of the states displayed in Figures 4-6. Structures of highlighted clusters are displayed in Figures 7 and S12 to emphasize the connection between the bent structures and distorted loop states (see text for detail).

| **10×AUUCU**  1 X-b-e-a-a-X-a-b  2 X-b-e-a-a-X-a-b  3 X-a-e-a-a-X-a-b  4 X-b-e-a-a-X-a-b  5 c-a-X-a-a-X-a-b  6 X-b-c-a-a-X-a-b  7 c-a-c-d-b-X-a-c  8 c-a-c-a-b-X-a-c  **9 c-b-c-d-b-X-a-c**  10 a-d-c-a-a-X-a-a  11 e-b-X-a-a-X-a-b  12 a-d-c-a-b-X-a-a  13 c-a-X-a-a-X-a-b  14 a-c-c-a-a-X-a-a  15 c-a-c-d-b-X-a-c  16 e-b-e-a-a-X-b-b  17 c-a-c-c-b-X-a-c  18 c-b-c-d-d-X-a-c  19 X-a-c-a-b-X-a-c  20 X-a-d-c-b-X-a-c | **15×CCUG**  1 a-a-d-d-a-a-a-a-a-a-a-a-a  2 a-a-f-d-a-a-a-a-a-d-a-d-d  3 a-a-a-d-a-a-a-a-a-a-a-a-X  4 a-a-d-d-a-a-a-a-d-d-a-a-d  5 a-a-f-d-a-a-a-a-a-d-a-d-d  6 a-d-d-d-a-a-a-a-d-a-a-f-d  7 X-a-d-d-a-a-a-a-a-a-a-a-a  8 d-d-f-d-a-a-a-a-d-d-a-d-d  9 d-d-d-f-a-a-a-a-d-d-a-d-d  10 d-d-d-f-a-a-a-a-d-d-a-f-d  11 X-a-d-d-a-a-a-a-a-a-a-a-a  12 a-a-a-d-a-a-a-a-a-a-a-a-a  13 a-d-d-d-a-a-a-f-X-d-a-d-d  14 a-a-d-d-a-a-a-a-f-f-a-d-d  15 a-a-a-a-a-a-d-a-a-a-a-a-X  16 X-a-d-f-a-a-a-a-a-a-a-a-d  17 a-a-a-a-a-a-a-a-a-a-a-a-a  18 a-a-f-d-a-a-a-a-a-a-a-f-d  **19 d-d-d-d-a-a-a-a-d-d-a-d-d**  20 X-d-f-f-a-a-a-d-d-d-d-f-d |
| --- | --- |
| **20×CAG**  1 a-a-a-X-a-a-a-a-a-a-a-a-X-a-b-a-a-a  2 a-a-X-a-a-a-a-a-a-a-a-a-X-a-b-a-a-a  3 a-b-X-a-a-a-a-a-a-a-a-a-X-a-b-a-a-a  4 a-a-a-a-a-a-a-a-a-a-a-a-X-a-b-a-a-a  5 a-a-a-b-X-a-a-a-X-b-a-a-a-a-b-a-a-a  6 a-a-X-a-a-a-a-a-a-a-a-a-X-a-b-a-a-a  7 a-b-b-a-a-a-a-a-a-a-a-a-X-a-b-a-a-a  8 a-a-X-a-a-a-X-X-a-a-a-a-a-a-b-a-a-a  9 a-a-a-a-a-a-X-X-a-a-a-X-X-a-b-a-a-a  10 X-X-a-b-a-a-a-a-a-a-a-a-a-a-a-a-a-a  11 a-a-a-a-X-a-a-a-a-b-b-a-a-a-b-a-a-a  12 a-b-a-a-a-a-a-a-a-a-a-a-X-a-b-a-a-a  13 a-a-a-b-X-X-a-a-a-a-X-X-b-a-b-a-a-a  14 a-a-a-a-a-a-a-a-a-a-X-X-X-a-b-a-a-a  15 a-a-a-b-X-a-a-a-b-b-b-a-a-a-b-a-a-a  16 a-a-a-a-a-a-a-a-a-a-a-X-X-a-a-X-X-X  **17 a-a-a-a-b-a-a-a-a-b-b-a-a-a-b-a-a-a**  18 a-b-a-a-a-a-X-X-a-a-a-a-a-a-b-a-a-a  19 a-a-a-a-X-X-a-a-a-a-X-X-b-a-b-a-a-a  20 X-X-a-b-b-X-X-X-X-X-X-b-b-X-a-a-X-b | **20×CUG**  1 f-f-f-f-f-f-f-f-f-f-f-c-c-c-c-c-X-X  2 c-f-f-f-f-f-f-f-c-c-f-c-c-f-f-f-c-c  3 c-f-f-f-f-f-f-f-f-f-f-c-f-f-c-f-f-c  4 c-f-f-f-c-f-f-f-f-c-f-f-c-c-f-f-c-c  5 f-f-c-c-f-f-f-f-f-f-f-f-f-f-c-f-f-c  6 f-f-c-f-f-f-f-f-f-f-f-f-f-f-f-f-f-f  7 f-f-c-c-f-f-c-c-c-c-c-f-c-c-c-c-c-c  8 c-f-c-f-f-f-c-f-f-c-f-f-f-f-c-f-f-c  9 f-f-e-c-f-f-e-c-f-f-c-c-f-f-c-c-f-c  10 f-c-c-f-f-f-f-c-c-f-e-c-c-c-c-c-f-f  11 c-e-c-c-f-e-e-c-c-c-c-f-c-c-c-f-f-c  12 c-f-f-f-f-f-f-f-f-f-f-f-f-f-f-f-f-c  13 f-f-c-f-c-f-f-f-c-c-f-c-c-c-c-f-f-c  14 f-f-f-c-f-f-e-c-f-f-c-c-f-f-f-c-f-c  15 c-f-f-e-e-f-f-c-c-f-f-c-f-f-f-f-f-c  16 c-c-e-f-c-f-c-f-f-c-f-f-f-c-c-e-e-f  17 f-e-e-f-f-f-f-c-f-f-f-f-f-f-c-c-f-f  18 c-c-f-f-c-f-f-f-c-f-f-c-c-c-f-c-c-e  **19 c-c-e-e-e-e-c-f-d-f-f-f-f-f-f-d-f-f**  20 f-c-c-e-e-f-f-c-c-f-e-c-c-f-f-c-f-f |


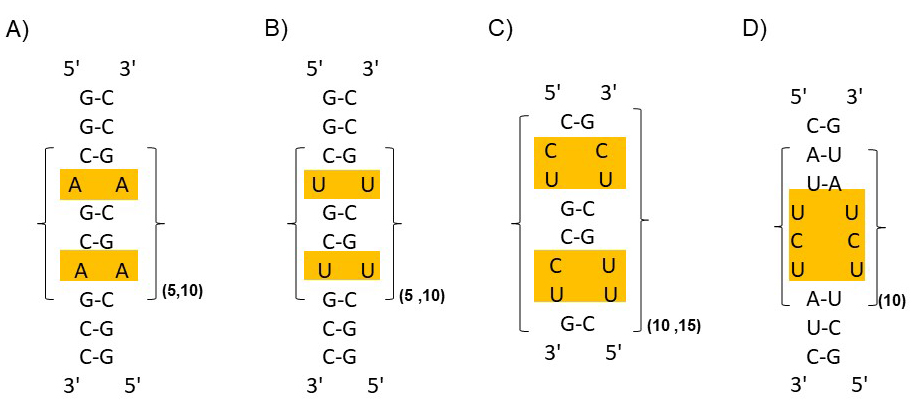


**Figure S1.** Secondary structures of expanded repeats studied. The numbers inside the parenthesis display the number of repeats (see also Table 1).





**Figure S2.** Bending angle (**°**) as a function of time calculated for 20×CUG (A), 20×CAG (B), 10×CAG (C), 10×CUG (D), 15×CCUG (E), 10×CCUG (F) and 10×AUUCU (G). The plots show changes of the bending angle of the RNA systems along the MD trajectory. Note that in A, B, E, and G, which are the biggest RNA systems investigated, bending angles fluctuates a lot, as described in the main article.


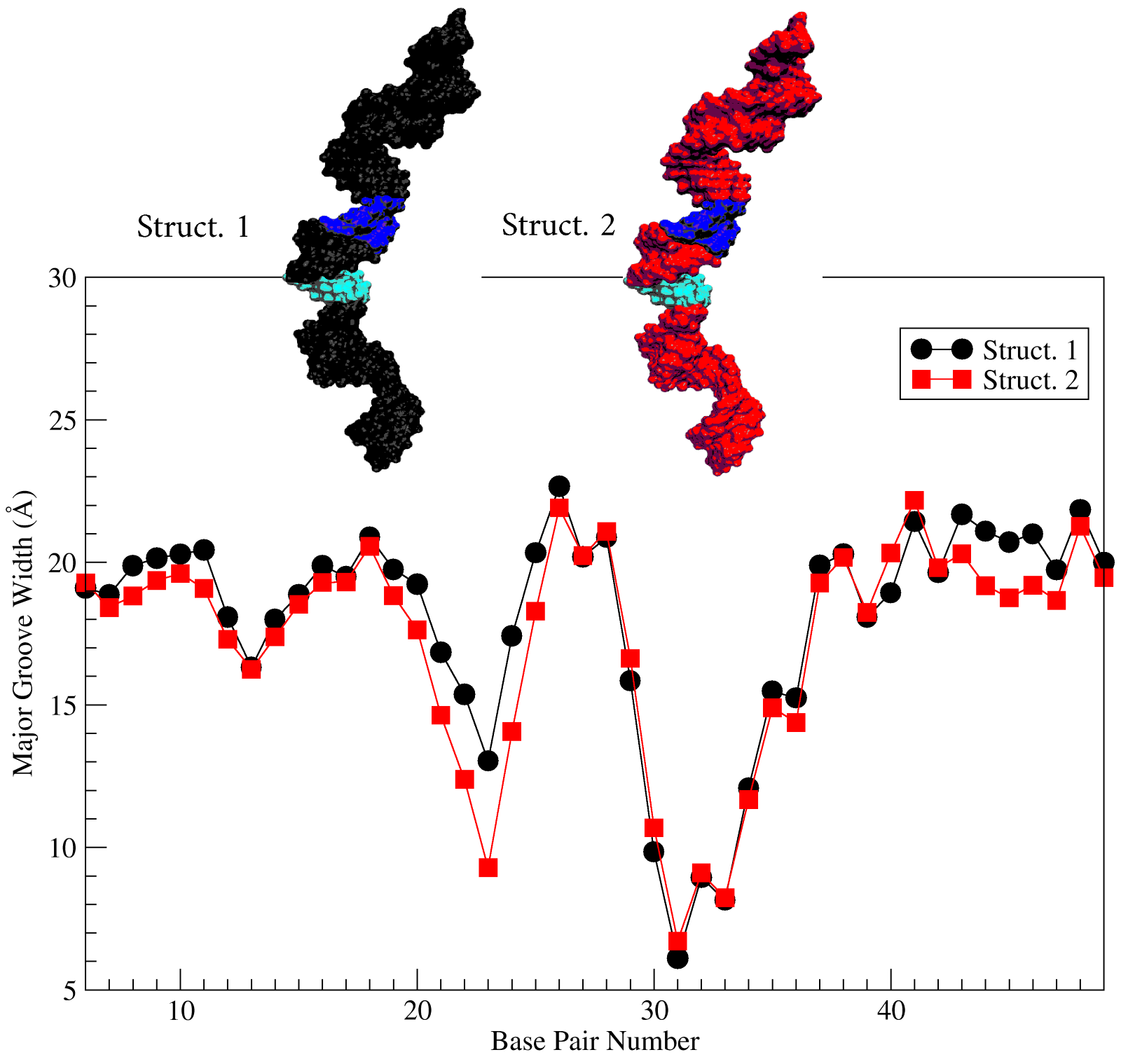


**Figure S3**. Major-groove width (Mgw) analyses performed on the two most bent states observed in 10×AUUCU with structures 1 and 2 highlighted in black and red, respectively. First five base pairs at each terminal are excluded from the analyses. Around base pair step # 23 highlighted in blue, Mgw shrinks to ~13 Å and ~10 Å in structures 1 and 2, respectively. Another drastic change in the Mgw is observed around base pair step # 31 highlighted in cyan where Mgw < 7Å.


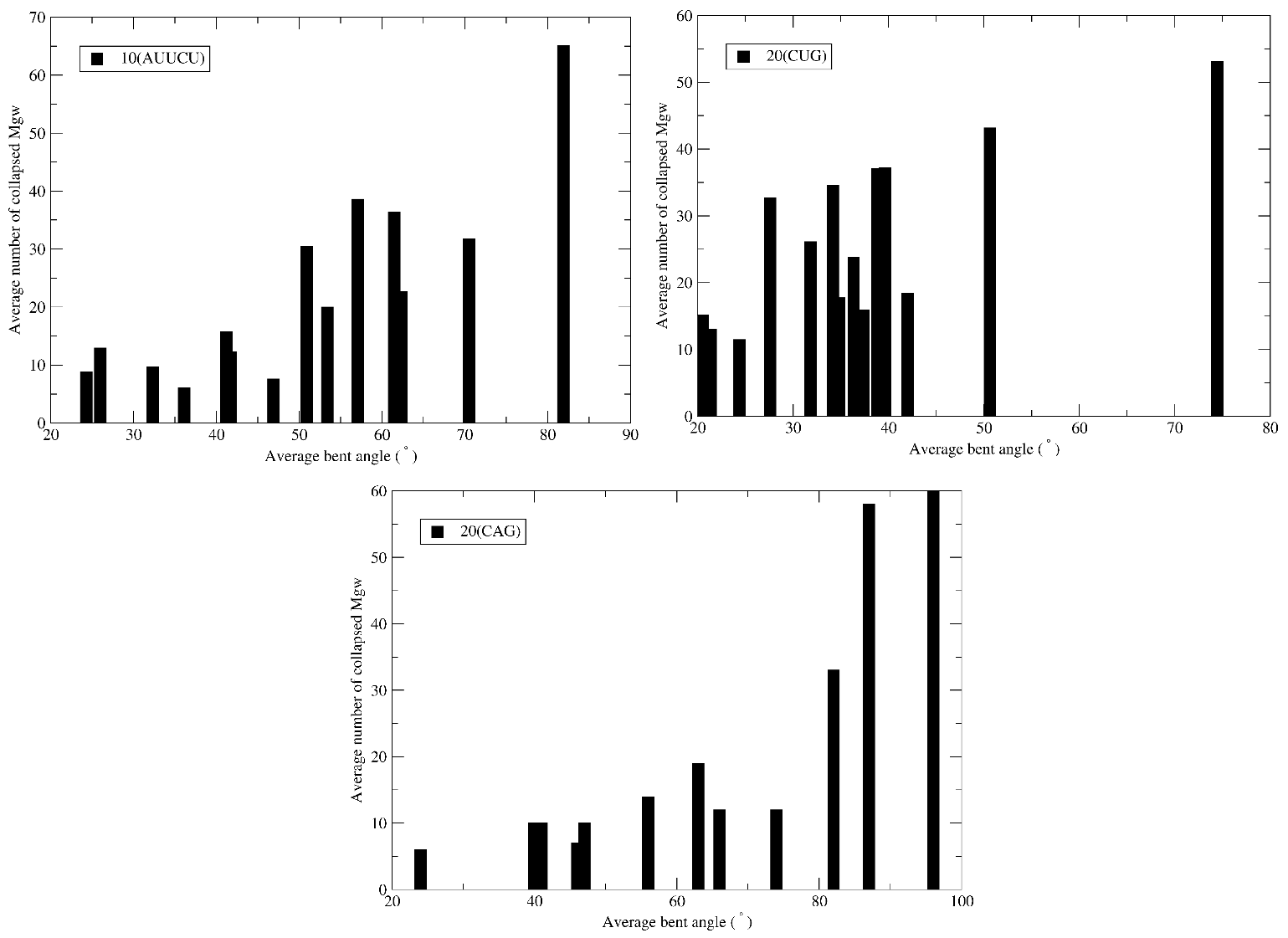


**Figure S4**. Average bending angle vs average number of collapsed Mgw observed in the clustered states of 10×AUUCU, 20×CUG, and 20×CAG. Collapse of Mgw is defined as a value < 18 Å. Note that as the bending angles increase, the total number of collapsed Mgw along RNA increase, too.


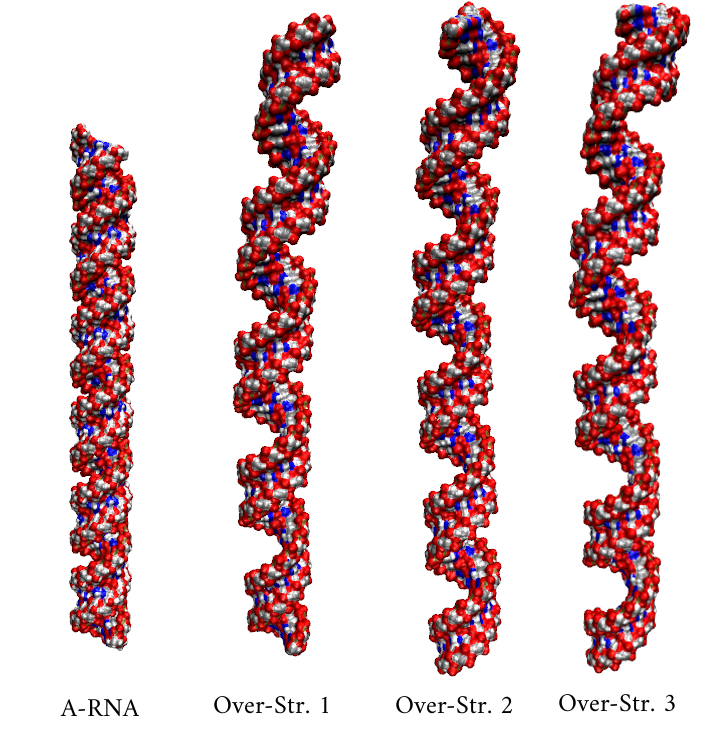


**Figure S5.** Average structures of overstretched conformations observed in the 20×CUG (Table S2). A-form RNA is given as a reference for better comparison with over-stretched conformations.


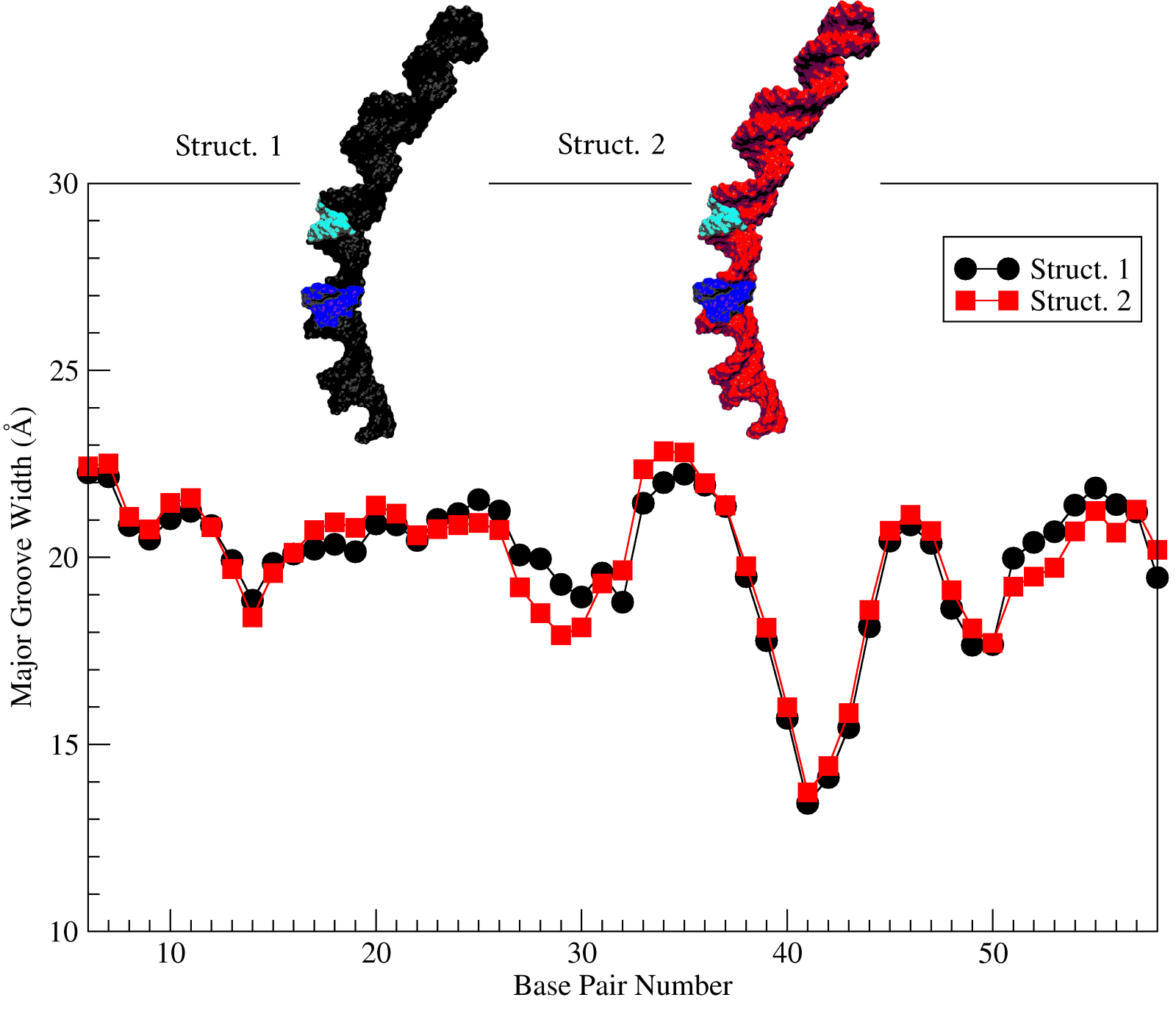


**Figure S6.** Major-groove width analyses of the two most bent states observed in 20×CUG. As a general pattern, also observed in 10×AUUCU, collapse of Mgw is the reason for bending and observed curvatures. Around base pair step # 41 highlighted in blue, Mgw decreases from an average value of ~20 Å to ~14 Å. A less pronounced change in the Mgw also happens around base pair step # 29 highlighted in cyan where Mgw drops to ~18 Å.


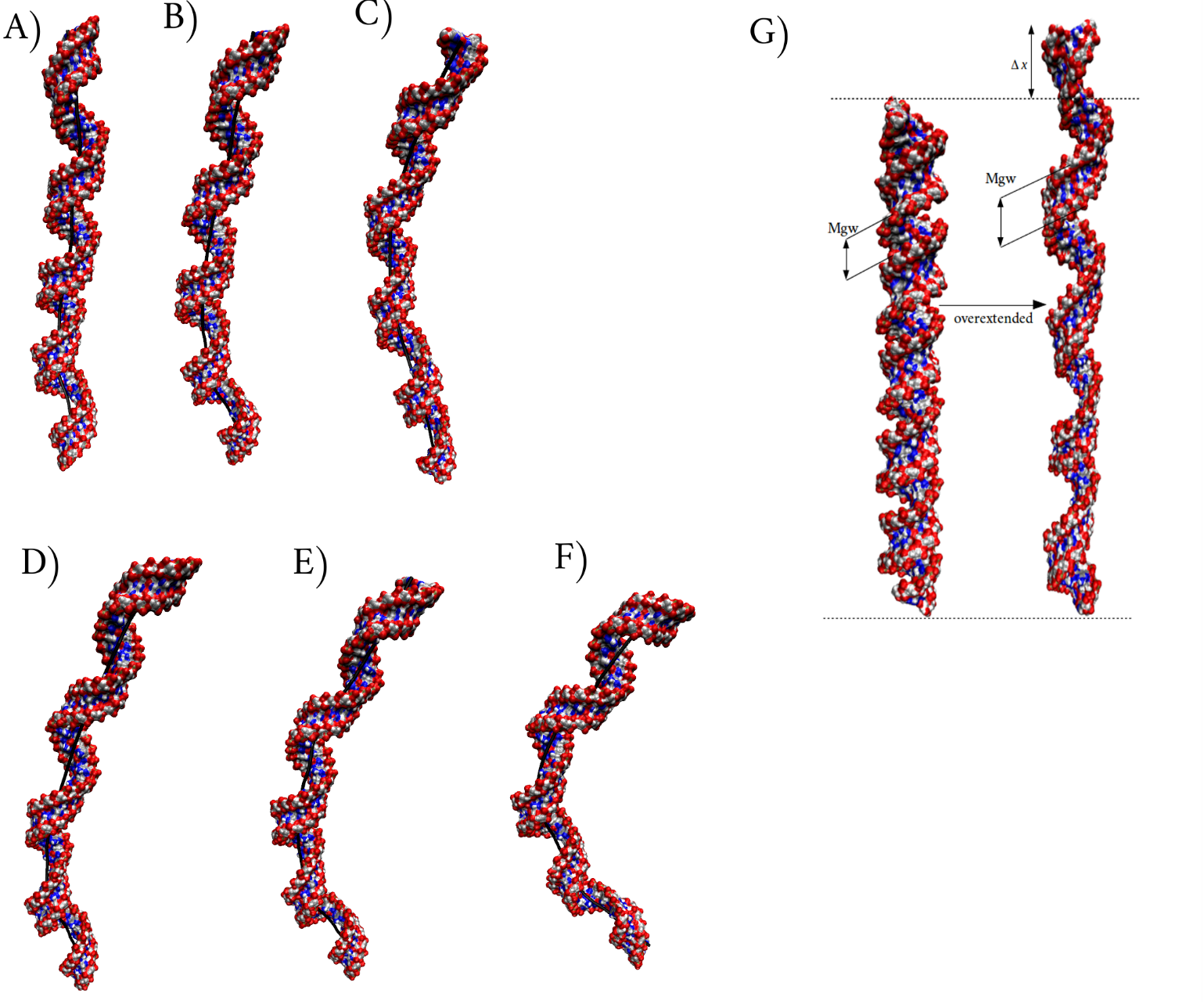
**Figure S7.** Bending (A-F) and over-extension (G) observed in 20×CAG. Each structure in A-F represents different bent states displayed in Table 2. The curvilinear helical axis is shown as a bold black line to emphasize the curvature in each case. While average rise value in relaxed structure is 2.8 Å, over-extended conformation has average rise value around 3.4 Å due to increase in major groove width.


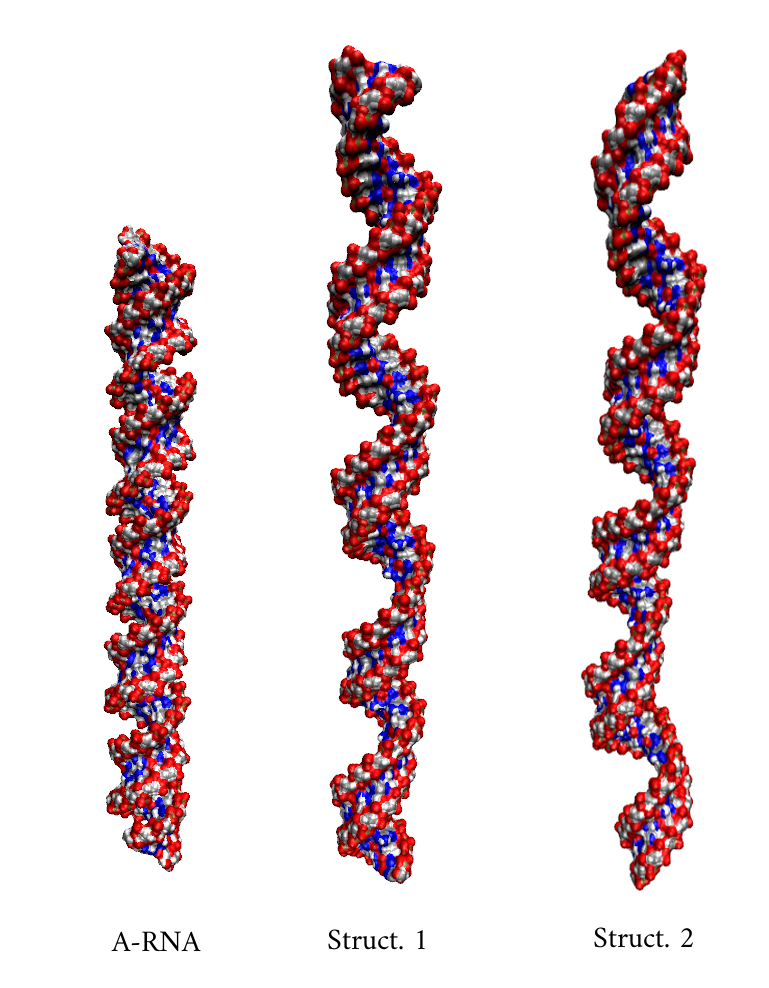


**Figure S8.** Average structures of overstretched conformations observed in the 20×CAG (Table S2). A-form RNA is given as a reference for better comparison with over-stretched conformations.


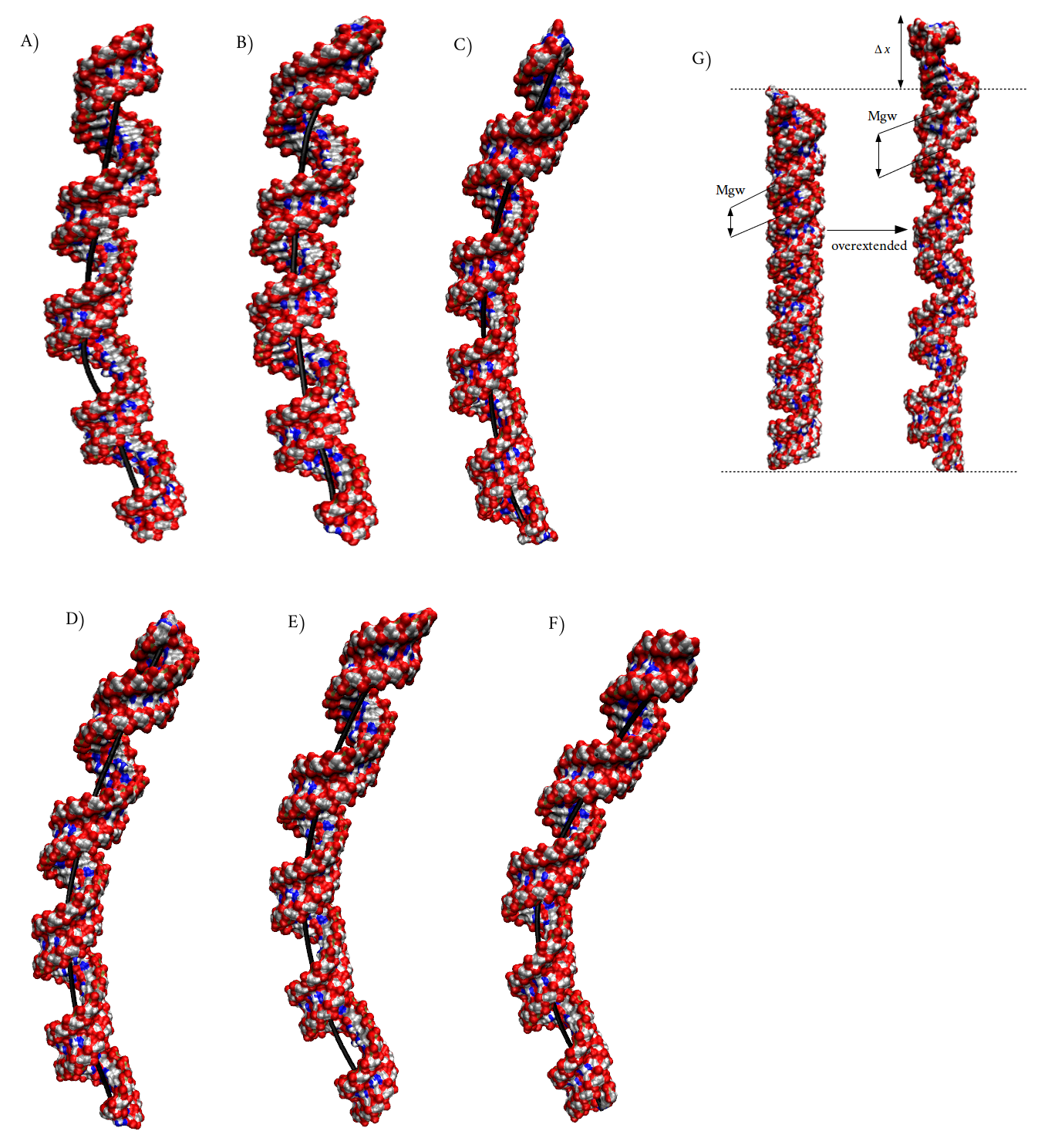


**Figure S9.** Bending (A-F) and over-extension (G) observed in 15×CCUG. Each structure in A-F represents different bent states displayed in Table 2. The curvilinear helical axis is shown as a bold black line to emphasize the curvature in each case.


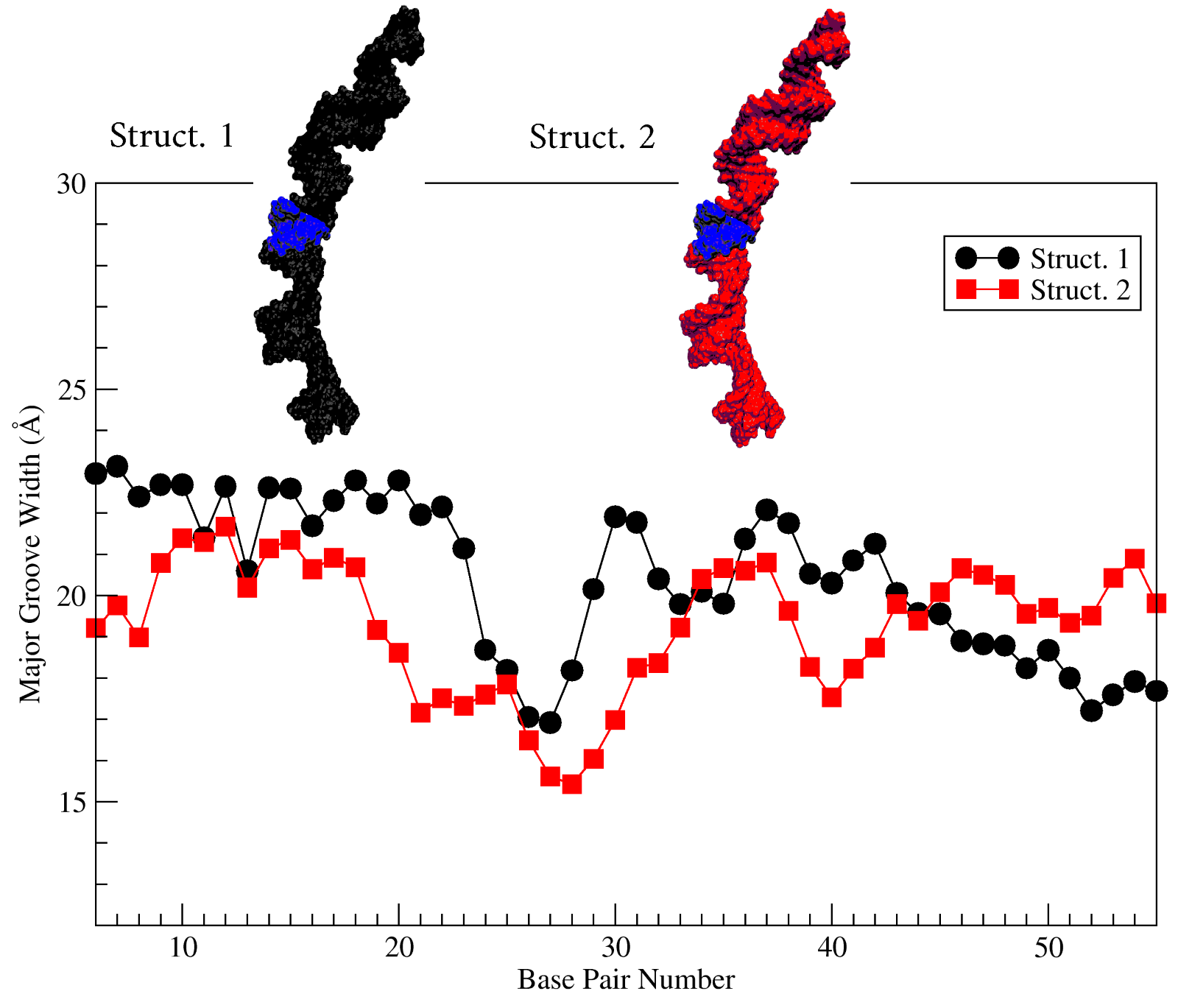


**Figure S10.** Major-groove width analyses of the two most bent states observed in 15×CCUG. Like the other systems Mgw is responsible for the observed curvatures. Despite other systems, changes of Mgw in 15×CCUG are less pronounced causing the system to manifest moderate bending. A change in Mgw around base pair step # 25 highlighted in blue from ~20 Å to < 17 Å create bent conformations.

| **A) (CAG)_20_**  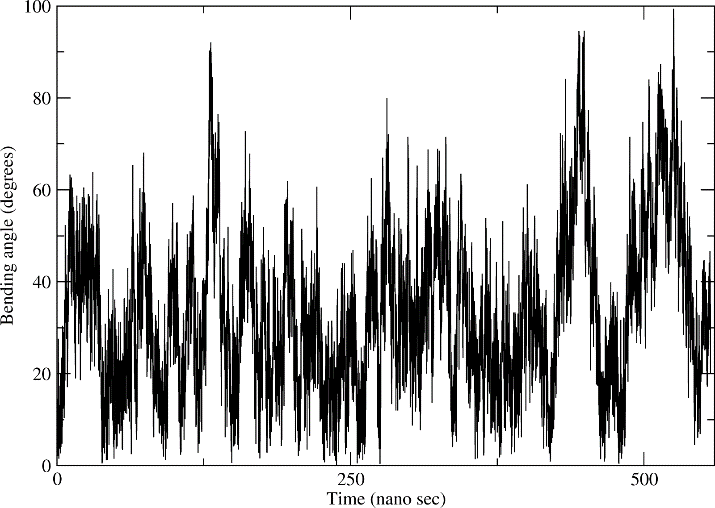 | **B) (CUG)_20_**  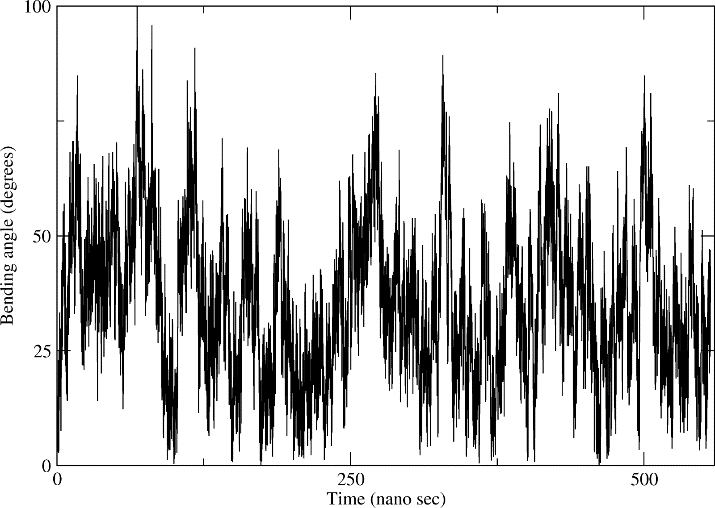 |
| --- | --- |
| **C) (AUUCU)_10_**  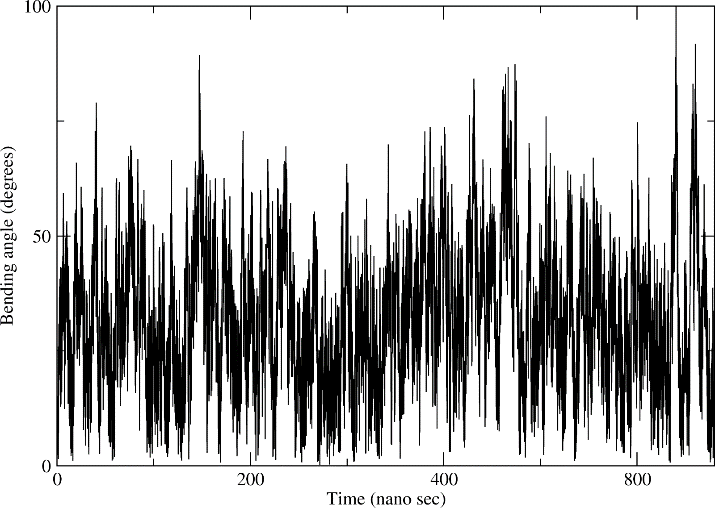 | **D) (CCUG)_15_**  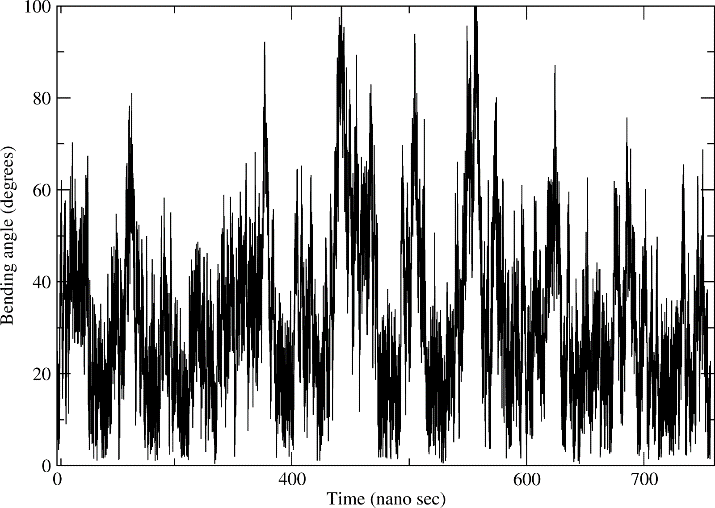 |

**Figure S11.** Bending angle (**°**) as a function of time calculated for fully Watson-Crick base paired 20×CAG (A), 20×CUG (B), 10×AUUCU (C) and 15×CCUG (D). The plots show changes of the bending angle of the RNA systems along the MD trajectory.


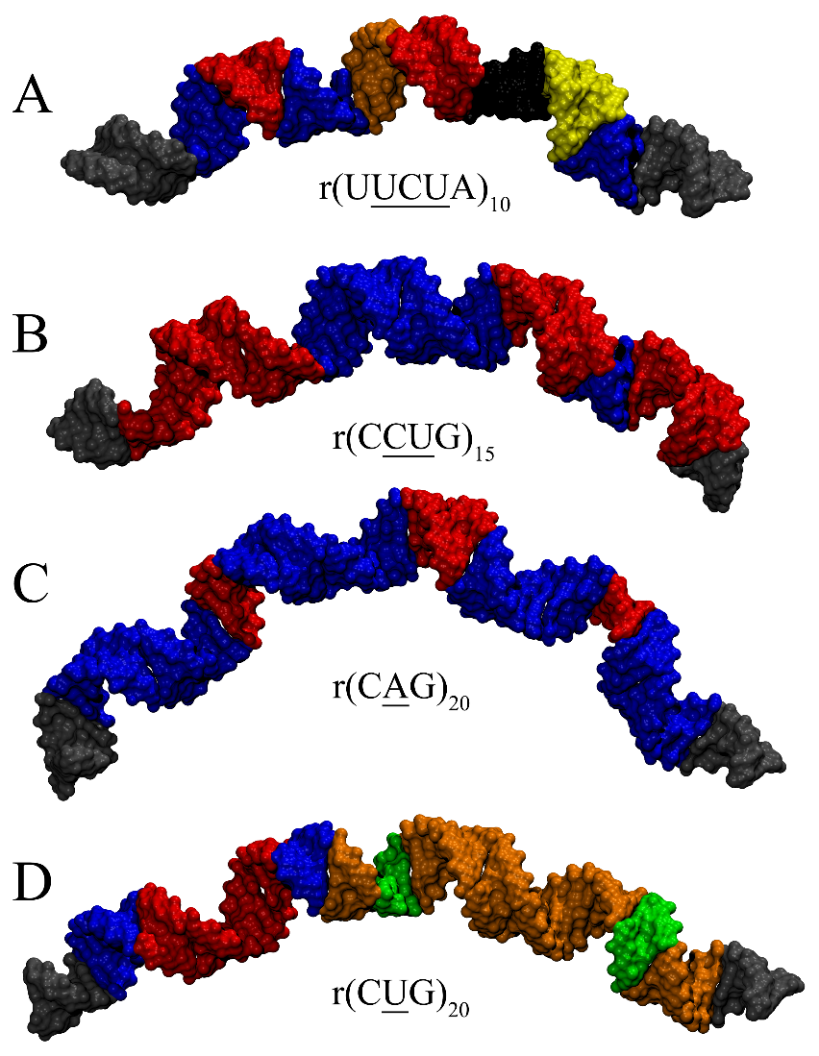


**Figure S12.** Loop conformations observed in representative clusters having extreme bending in (A) 10×AUUCU, (B) 15×CCUG, (C) 20×CAG, and (D) 20×CUG. Structures highlighted in Table S4 are displayed in the figure. Different colors are used to underline unique loop conformations displayed in Figures 4-6. The first and last loops in each structure are highlighted in gray, which are not compared to unique loop conformations determined. Loops highlighted in black could not be described with any clusters. In A, yellow, red, blue, and orange represent loop conformations of a, b, c, and d, respectively, displayed in Figure 4, where c and d have distorted closing base pairs. In B, blue and red represent loop conformations of a and d, respectively, displayed in Figure 5, where d has distorted closing base pairs. In C, blue and red represent loop conformations of a and b, respectively, displayed in Figure 6, where a represents 1×1 A/A loops with 0 hydrogen bonds. In D, blue, green, red, and orange represent loop conformations of c, d, e, and f, respectively, displayed in Figure 6, where e has distorted closing base pairs.


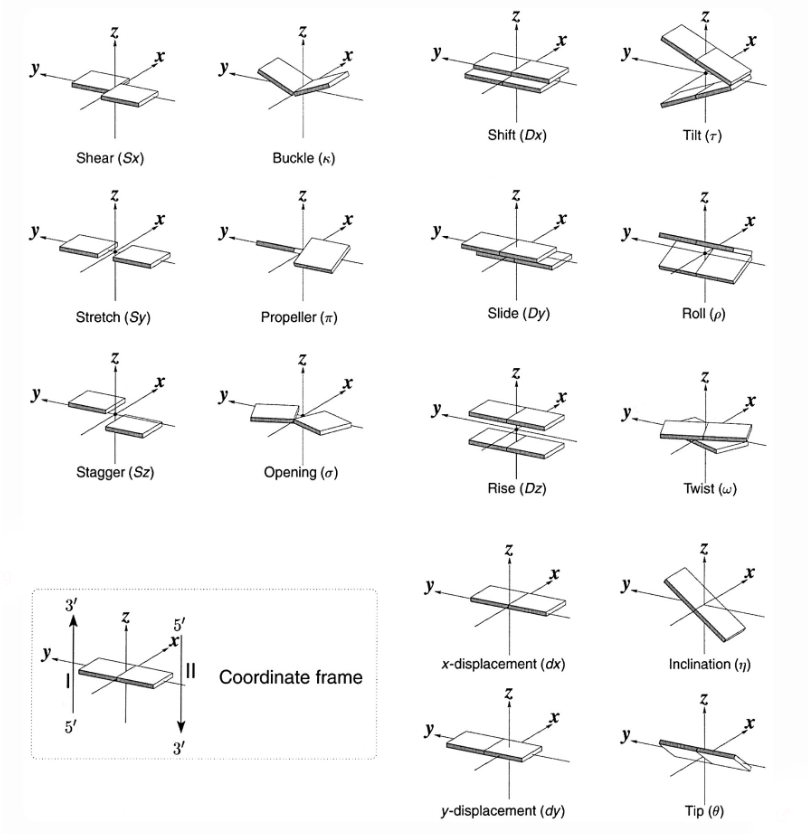

**Figure S13.** Schematic diagram of base pair and base step parameters.


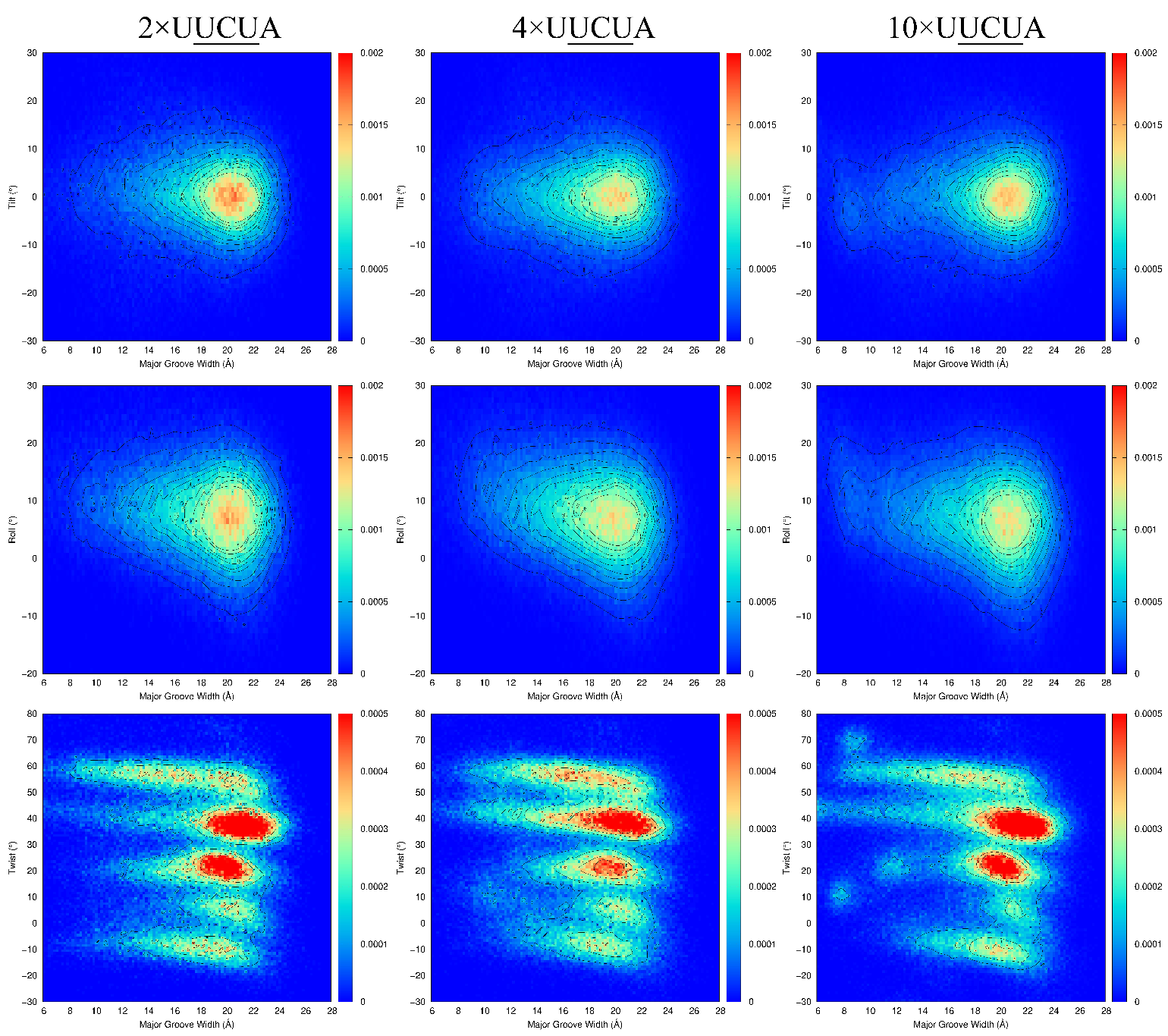


**Figure S14.** 2D distribution analyses performed on 2×AUUCU, 4×AUUCU, and 10×AUUCU to investigate correlation between major groove width (Mgw) and the base pair step angles (tilt, roll, and twist).


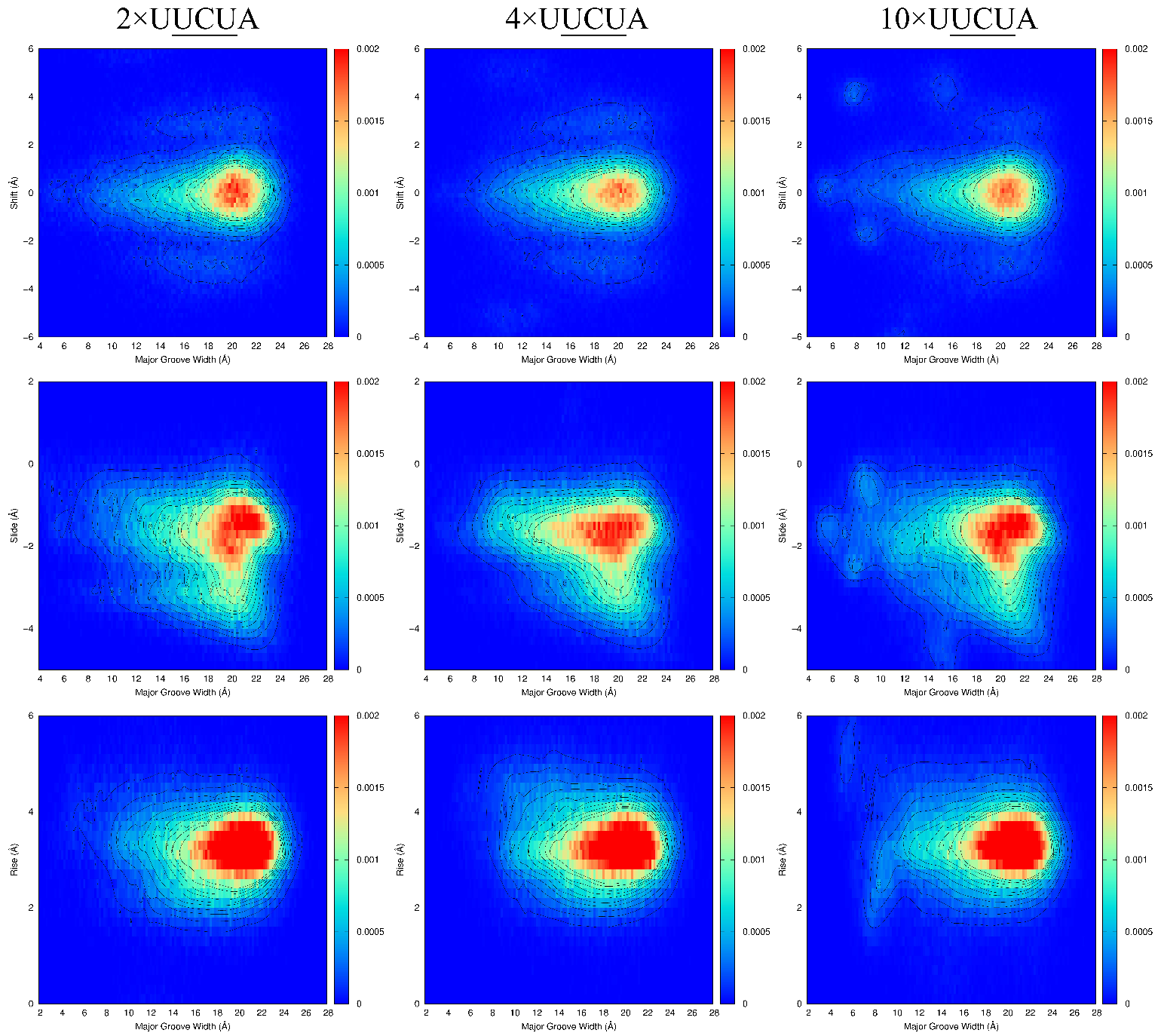


**Figure S15.** 2D distribution analyses performed on 2×AUUCU, 4×AUUCU, and 10×AUUCU to investigate correlation between major groove width (Mgw) and the base pair step distances (shift, slide, rise).


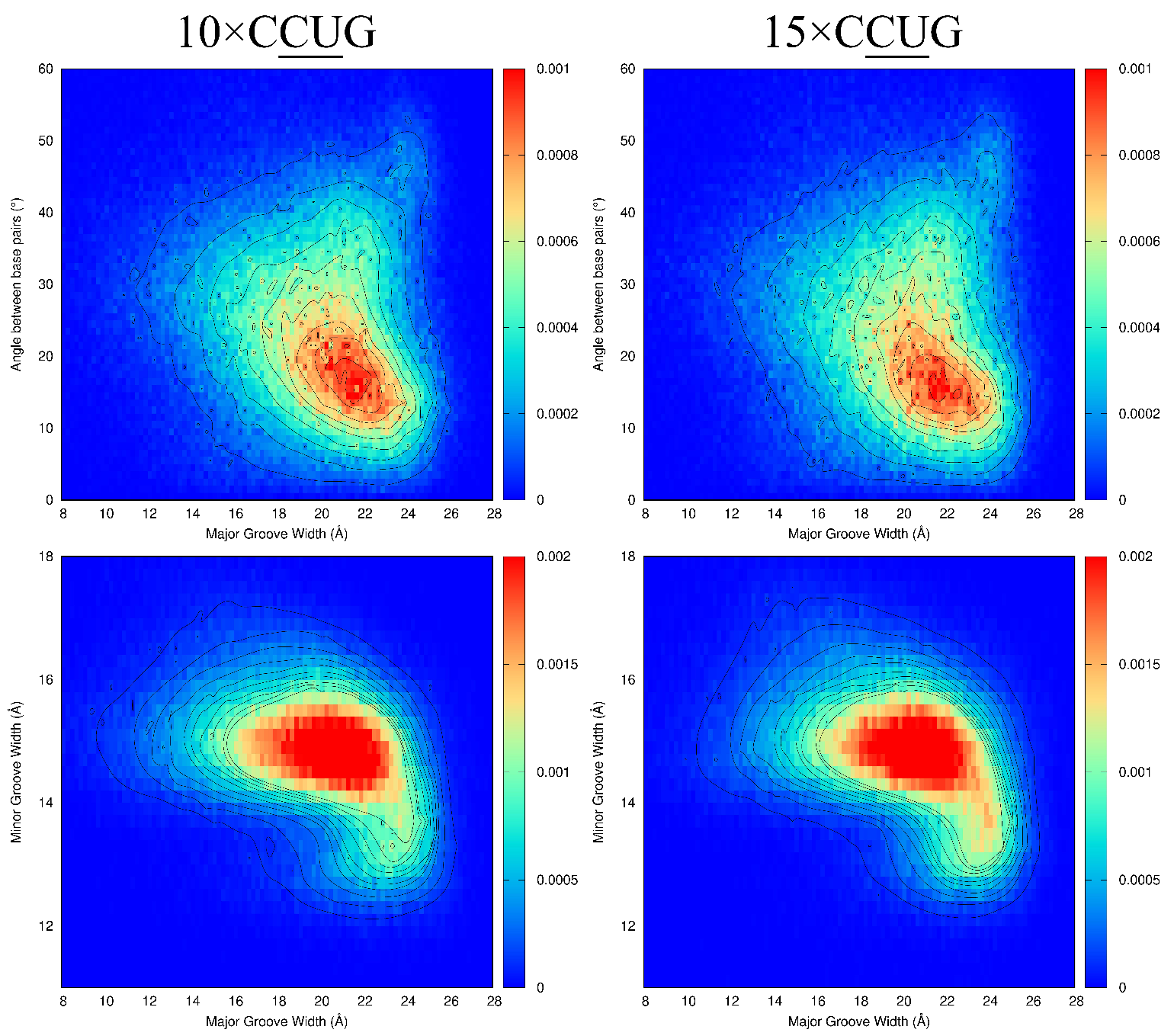
**Figure S16.** 2D distribution analyses performed on 10×CCUG and 15×CCUG to investigate correlation between major groove width (Mgw) and two parameters, a reaction coordinate, θ, representing the angular change of base-pair steps defined in Figure 9A and minor groove width (mgw).


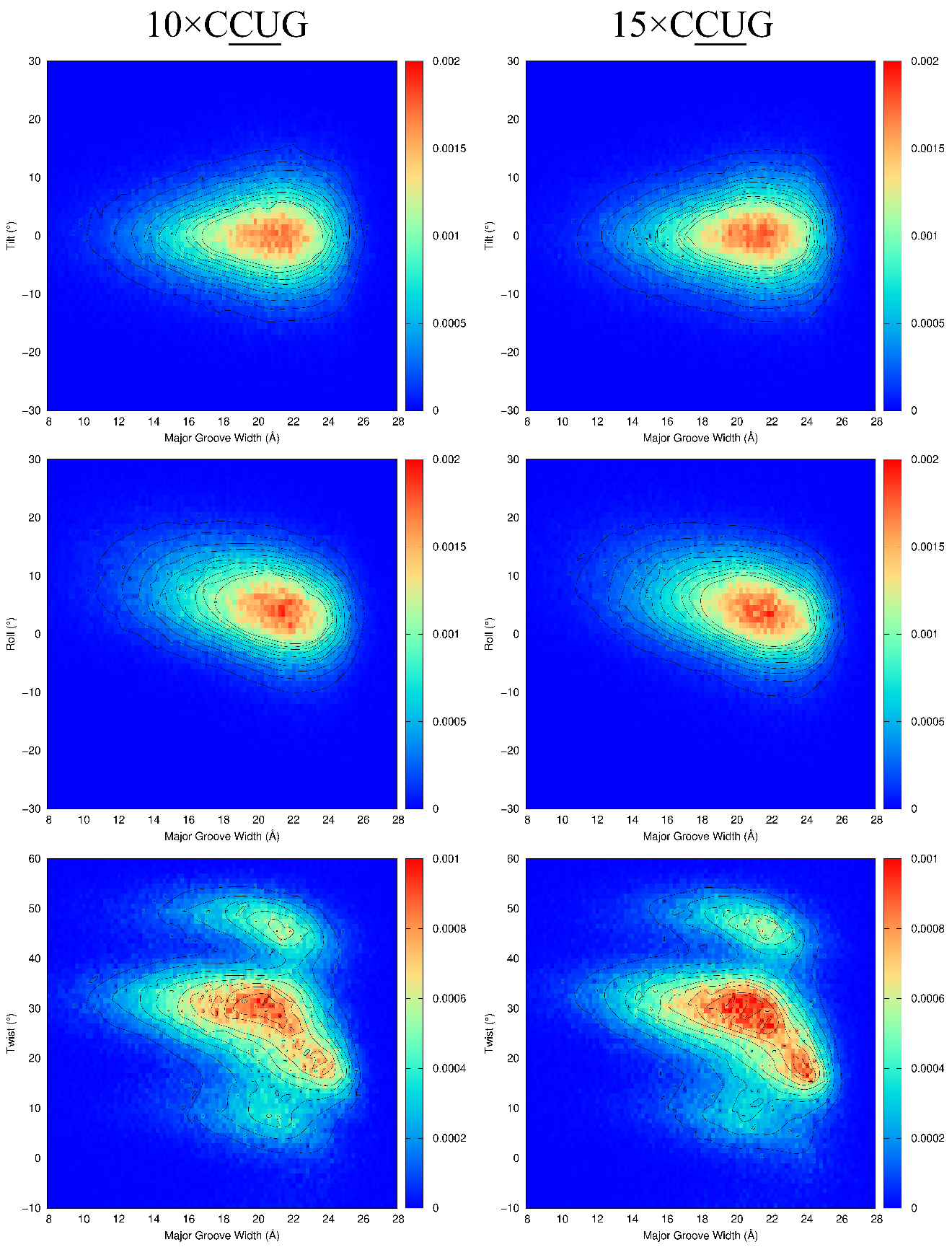


**Figure S17.** 2D distribution analyses performed on 10×CCUG and 15×CCUG to investigate correlation between major groove width (Mgw) and the base pair step angles (tilt, roll, and twist).


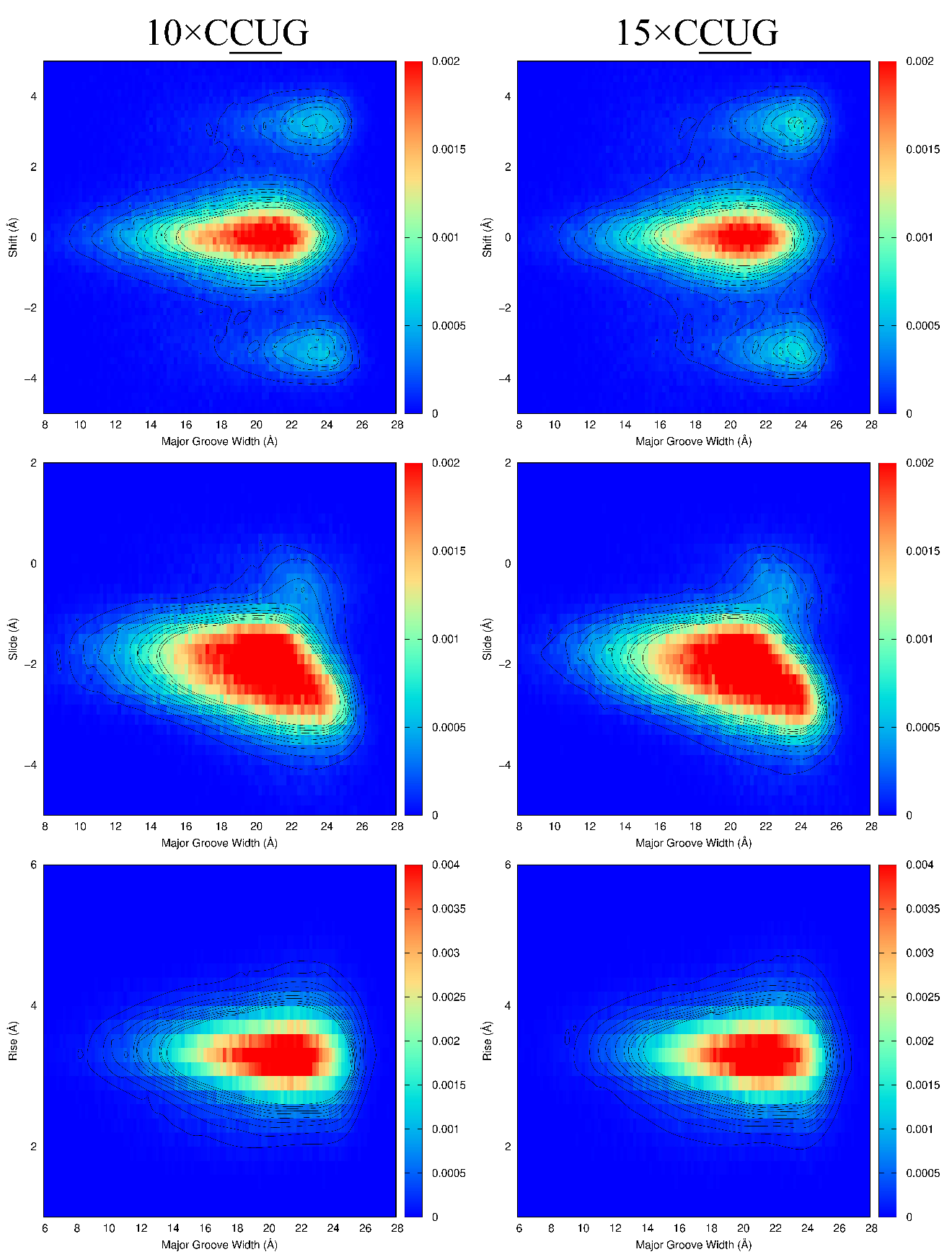


**Figure S18.** 2D distribution analyses performed on 10×CCUG and 15×CCUG to investigate correlation between major groove width (Mgw) and the base pair step distances (shift, slide, rise).


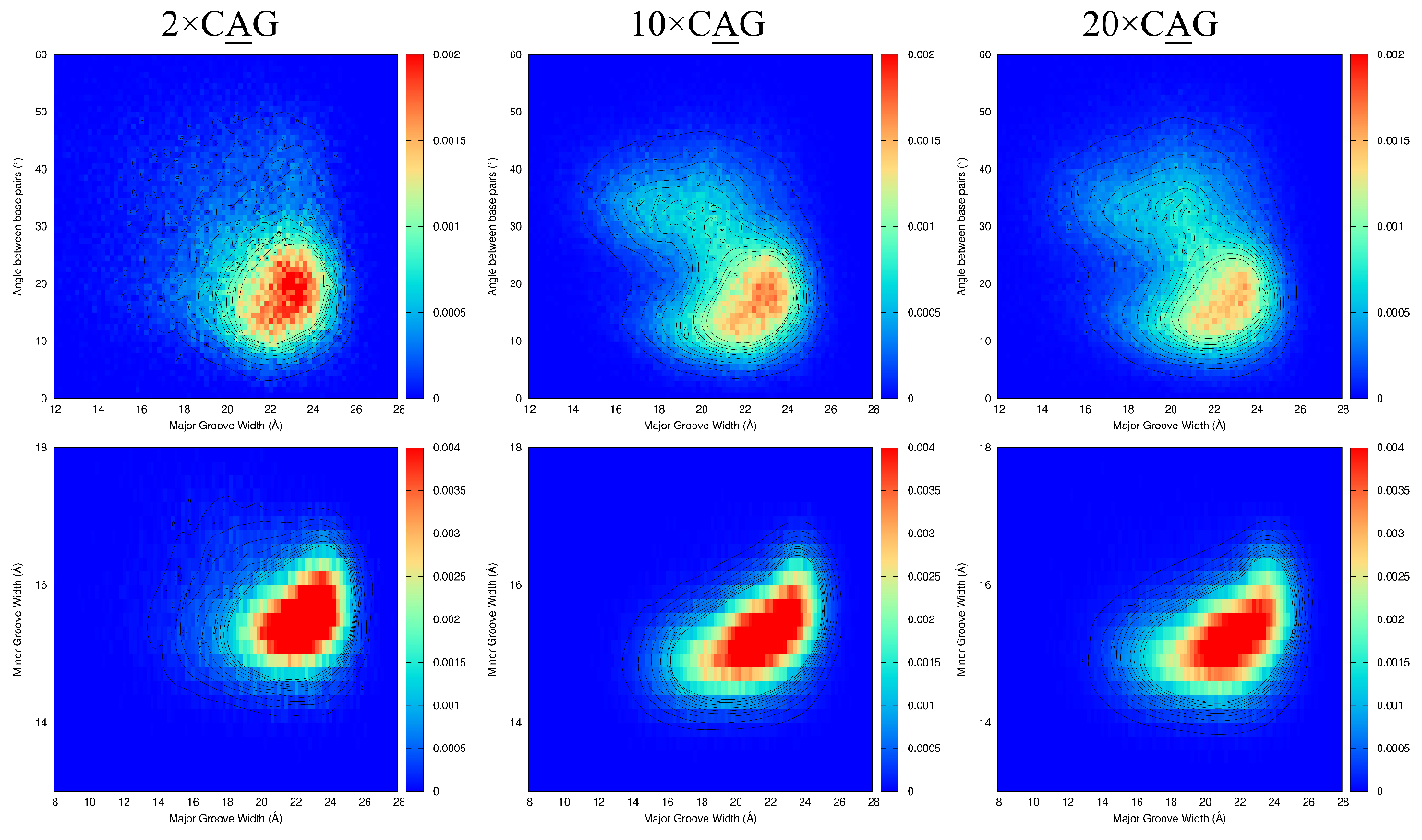


**Figure S19.** 2D distribution analyses performed on 2×CAG, 10×CAG, and 20×CAG to investigate correlation between major groove width (Mgw) and two parameters, a reaction coordinate, θ, representing the angular change of base-pair steps defined in Figure 9A and minor groove width (mgw). Note that as the repeat size is increased, θ ~ 35° with Mgw ~ 19 Å is sampled in 10×CAG and 20×CAG.


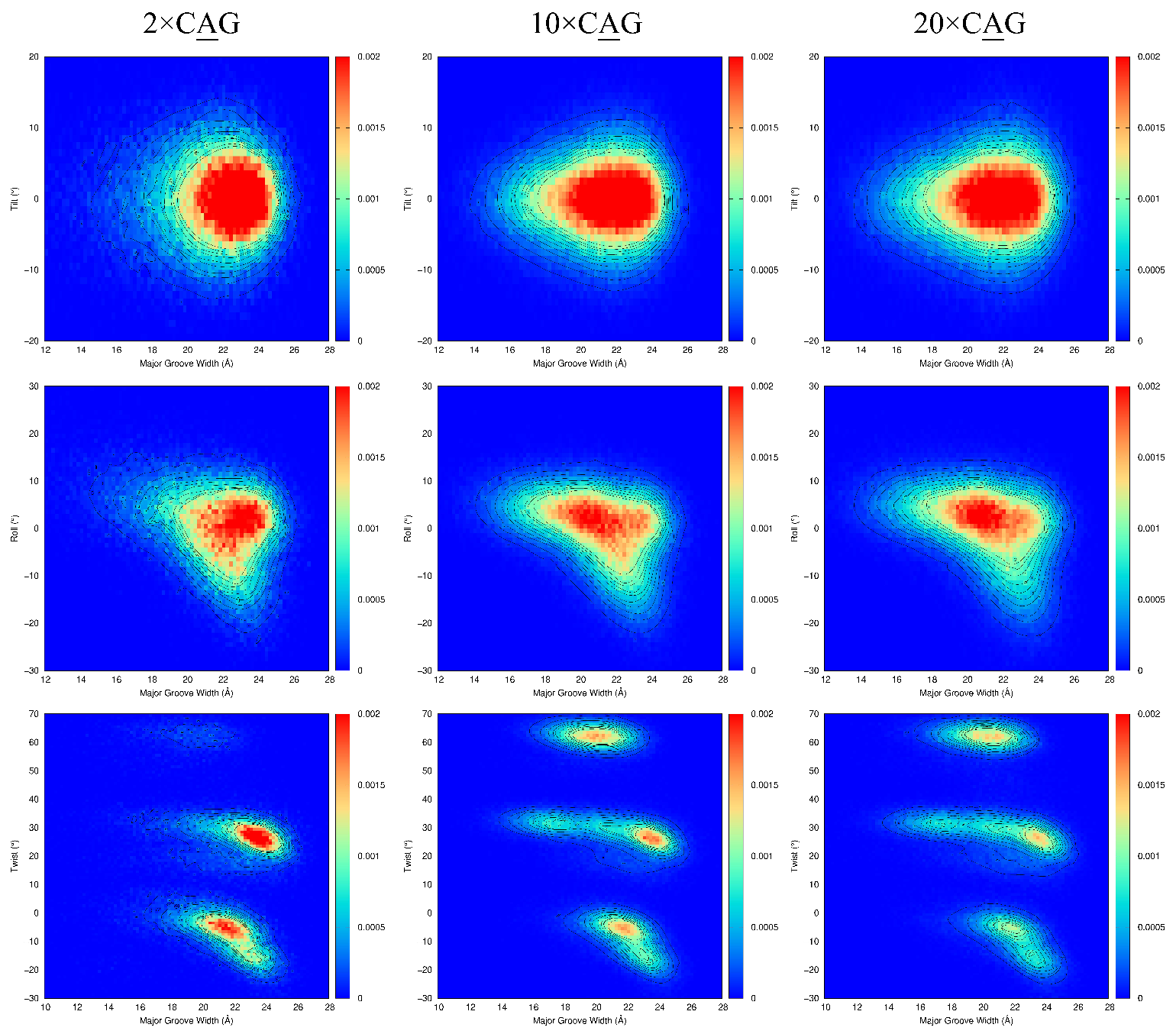


**Figure S20.** 2D distribution analyses performed on 2×CAG, 10×CAG, and 20×CAG to investigate correlation between major groove width (Mgw) and the base pair step angles (tilt, roll, and twist). Note that as the repeat size is increased, some states observed in 2×CAG such as twist ~ 5 ° with Mgw ~ 21 Å, and twist ~ 30 ° with Mgw ~ 24 Å is not sampled in 10×CAG, and 20×CAG. Note also that states with twist ~ 65 ° with Mgw ~ 20 Å is sampled more in 10×CAG and 20×CAG compared to 2×CAG.


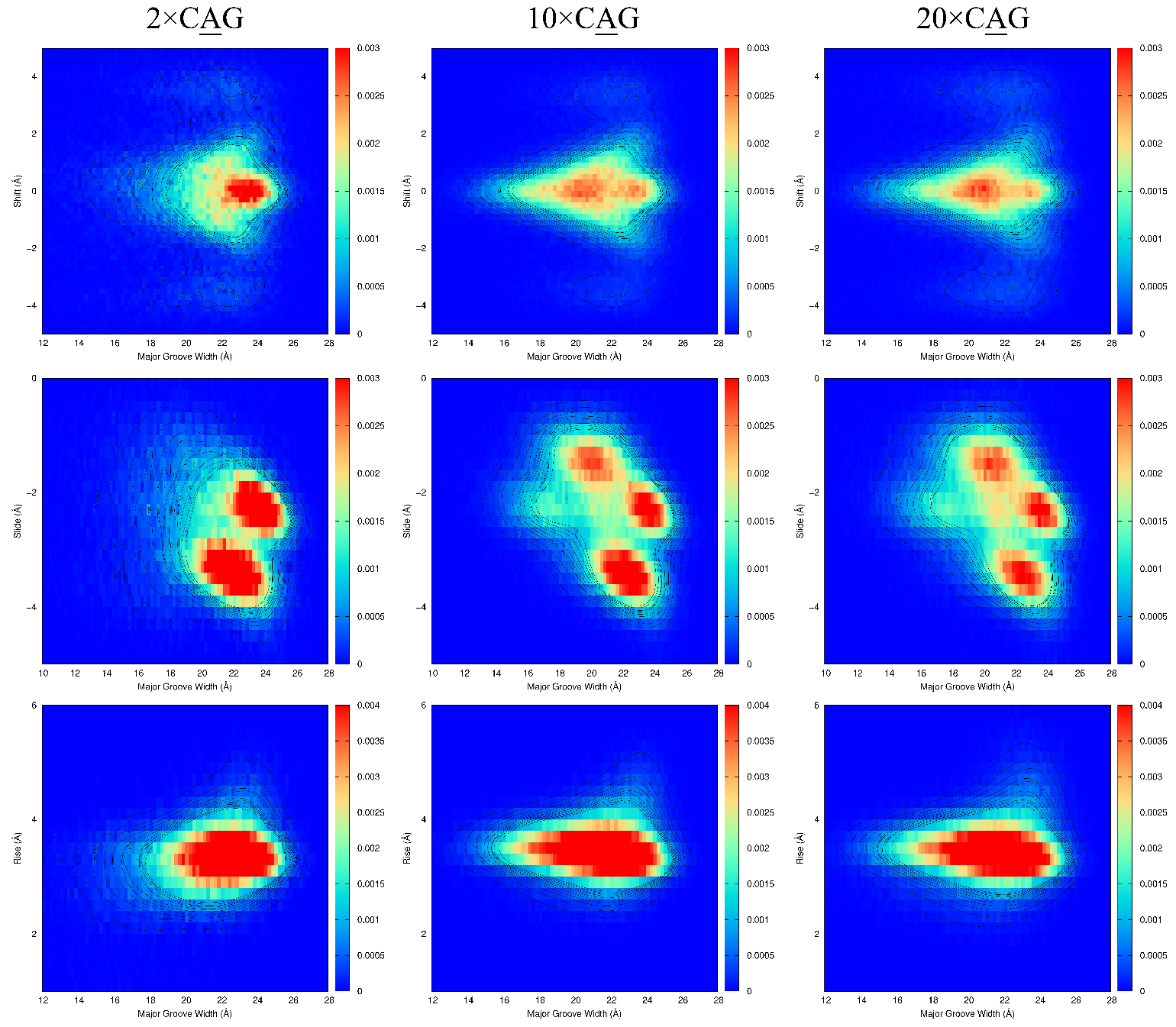


**Figure S21.** 2D distribution analyses performed on 2×CAG, 10×CAG, and 20×CAG to investigate correlation between major groove width (Mgw) and the base pair step distances (shift, slide, and rise). Note the differences observed in the 2D (Mgw, slide) distribution.


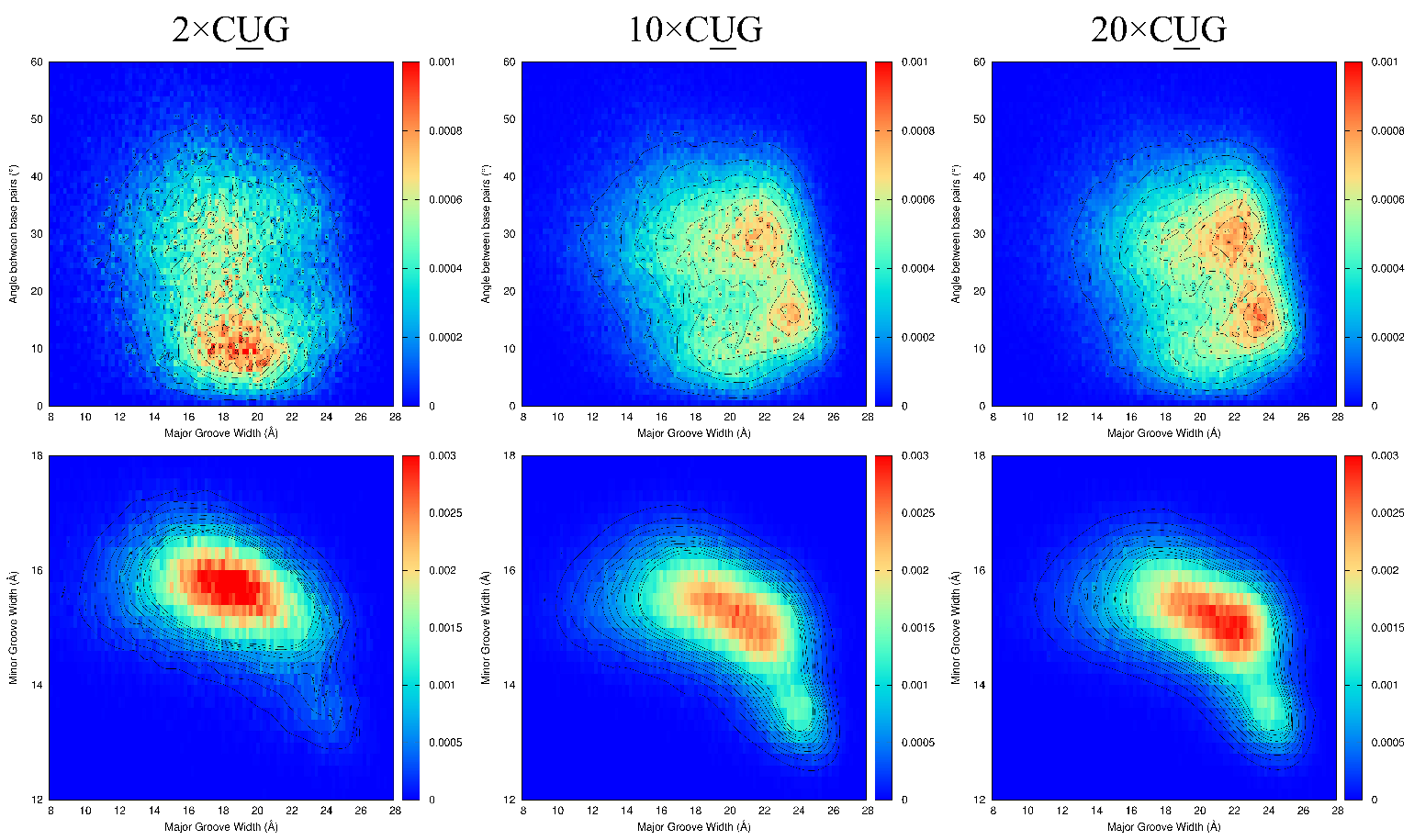


**Figure S22.** 2D distribution analyses performed on 2×CUG, 10×CUG, and 20×CUG to investigate correlation between major groove width (Mgw) and two parameters, a reaction coordinate, θ, representing the angular change of base-pair steps defined in Figure 9A and minor groove width (mgw). Note the shift in sampled conformations observed in 2D (Mgw, θ) distributions. Note also that the region, (Mgw=22 Å, mgw=13 Å), is sampled more in 10×CUG and 20×CUG.


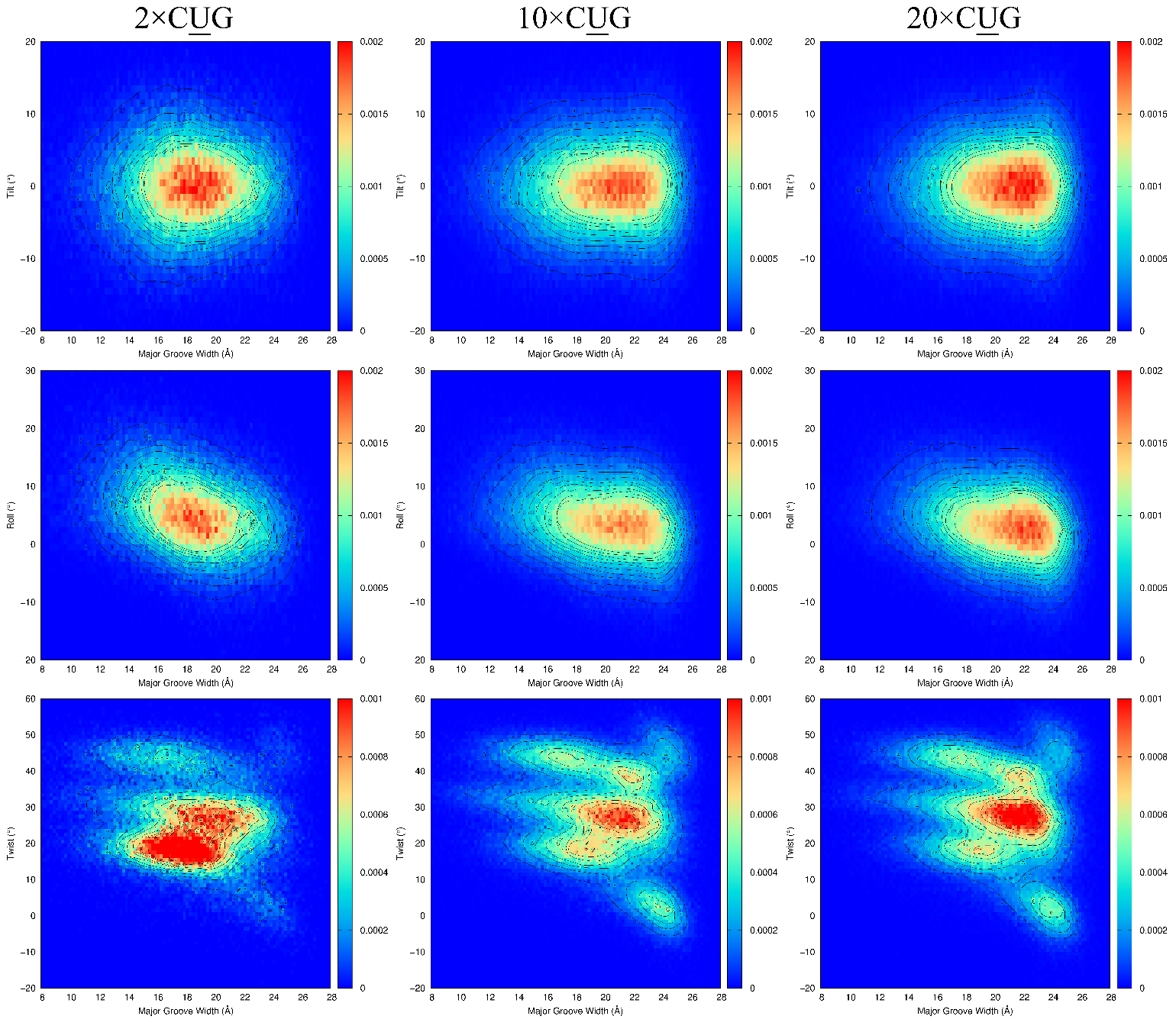


**Figure S23.** 2D distribution analyses performed on 2×CUG, 10×CUG, and 20×CUG to investigate correlation between major groove width (Mgw) and the base pair step angles (tilt, roll, and twist). Note the differences observed in 2D (Mgw, twist) distributions.


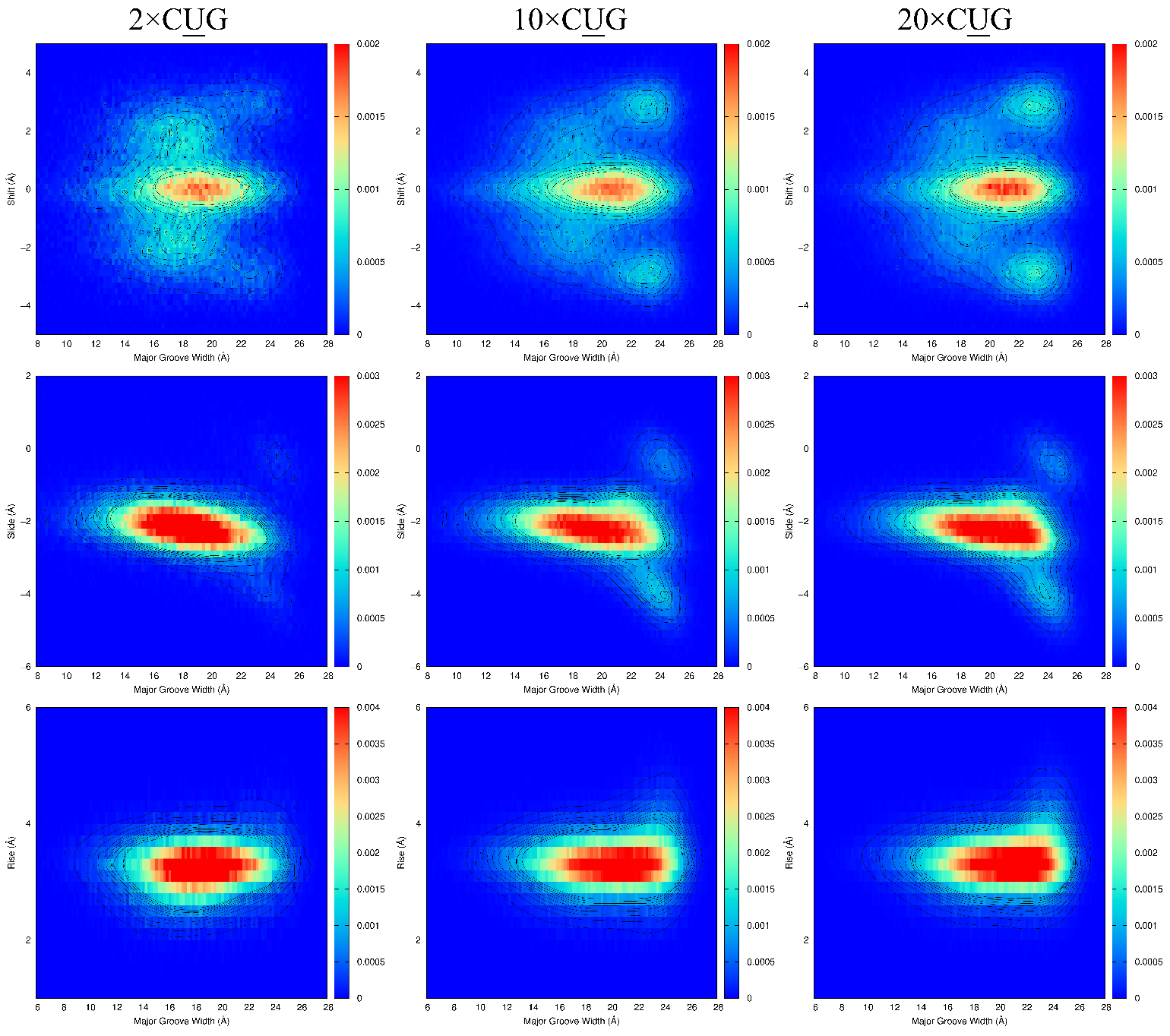


**Figure S24.** 2D distribution analyses performed on 2×CUG, 10×CUG, and 20×CUG to investigate correlation between major groove width (Mgw) and the base pair step distances (shift, slide, and rise). Note the differences observed in the 2D (Mgw, slide) and (Mgw, shift) distributions.
